# Development and characterization of silicone-based tissue phantoms for pulse oximeter performance testing

**DOI:** 10.1117/1.JBO.29.S3.S33314

**Published:** 2025-01-07

**Authors:** Anant Bhusal, Masoud Farahmand, Md Sadique Hasan, Sandhya Vasudevan, William C. Vogt, Bryan Ibarra, Sandy Weininger, Christopher G. Scully, X. Frank Zhang, Yu Chen, T. Joshua Pfefer

**Affiliations:** a University of Massachusetts, Department of Biomedical Engineering, Amherst, Massachusetts, United States; b Food and Drug Administration, Center for Devices and Radiological Health, Silver Spring, Maryland, United States

**Keywords:** pulse oximetry, arterial blood oxygen saturation, photoplethysmography, tissue phantoms, performance testing

## Abstract

**Significance:**

Pulse oximeter measurements are commonly relied upon for managing patient care and thus often require human testing before they can be legally marketed. Recent clinical studies have also identified disparities in their measurement of blood oxygen saturation by race or skin pigmentation.

**Aim:**

The development of a reliable bench-top performance test method based on tissue-simulating phantoms has the potential to facilitate pre-market assessment and the development of more accurate and equitable devices. To generate phantoms capable of mimicking physical mechanisms and providing realistic results, customized tissue-mimicking materials (TMMs) are needed.

**Approach:**

We focused on the development of channelized finger phantoms based on flexible silicone elastomers and their implementation in a pulsatile pressurized fluid network. Candidate TMMs were formulated to achieve a range of biologically relevant mechanical and optical properties by modifying components and curing protocols.

**Results:**

Our final optimized TMM had a Shore OO hardness of 32 and an elastic modulus of 130 kPa. TMM samples with sub-millimeter diameter channels exhibited compliance—increase in channel diameter with internal fluid pressure, as measured by optical coherence tomography—that was linearly dependent on internal pressure. Phantoms implemented in the pressurized network with an absorber-doped fluid and measured by a photoplethysmographic (PPG) sensor displayed tunable modulation levels ranging from 0.6% to 18.1% at 940 nm. Finally, we demonstrated that the system could be used to generate measurements in several clinical pulse oximeters and variations in PPG waveform could be produced by varying the simulated epidermal melanin content.

**Conclusions:**

Overall, we provide significant insights into potential best practices for creating silicone-based tissue phantom tools for pulse oximetry performance testing.

## 1 Introduction

Point-of-care monitoring of arterial blood oxygen saturation (${\mathrm{SaO}}_{2}$) is critical for managing patient health across a wide range of clinical scenarios. Among standard vital signs, oxygen saturation is regarded as the fifth essential indicator.^1^^,^^2^ However, recent retrospective studies have indicated that pulse oximeters—which display ${\mathrm{SpO}}_{2}$, a non-invasive estimate of ${\mathrm{SaO}}_{2}$—exhibit variations in performance correlated with race.^3^^,^^4^ Epidermal melanin is a dominant skin chromophore,^5^ known to impact optical signals at visible and near-infrared wavelengths.^6^ Race likely represents a surrogate for skin pigmentation in these clinical studies.^7^ In addition, recent clinical evidence links pulse oximeter bias with skin pigmentation.^8^ Such errors in ${\mathrm{SpO}}_{2}$ readings may result in delayed detection of hypoxemia and/or incorrect diagnosis.^9^

A pulse oximeter typically uses bands of red and infrared light (e.g., 660 and 940 nm) to generate a value (${\mathrm{SpO}}_{2}$) that approximates peripheral arterial ${\mathrm{SaO}}_{2}$. This measurement is based on differences in absorption coefficient ($\mu_{a}$) between oxygenated hemoglobin and deoxygenated hemoglobin at these wavelengths. Light from the pulse oximeter passes through biological tissues where it is subject to absorption and scattering. During systole, arterial blood volume content in the tissue increases, whereas during diastole, the tissue blood volume content decreases. The change in local blood volume over the cardiac cycle modulates the absorption of red and infrared light, resulting in a photoplethysmographic (PPG) waveform. This waveform can be broken down into a consistent “direct current” component due to non-vascular tissues (e.g., skin, fat, and bone) and a relatively stable background level of blood (especially venous) as well as a pulsatile “alternating current” component from the modulating arterial blood content. The quantity “percent modulation” (%Mod, sometimes referred to as pulsatility index) is calculated from the signal intensity at systolic and diastolic states ($I_{\text{sys}}$ and $I_{\text{dia}}$) as follows:

$$\%\mathrm{Mod}=\frac{I_{\mathrm{sys}}-I_{\mathrm{dia}}}{I_{\mathrm{dia}}}\times 100.$$ (1)

By evaluating this parameter at the red and near-infrared wavelengths, one can calculate a ratio of ratios (R).^10^

$$R=(\frac{{\%\mathrm{Mod}}_{\mathrm{red}}}{{\%\mathrm{Mod}}_{\mathrm{NIR}}}).$$ (2)

Pulse oximeters are typically calibrated empirically using measurements of R and corresponding CO-oximetry measurements in clinical studies.

Researchers have identified a number of factors that may help explain the correlation between melanin content and positive bias in ${\mathrm{SpO}}_{2}$ measurements. Clinical study results have indicated that pigmentation-correlated bias tends to arise for low $M_{f}$ levels, e.g., 1.0 or less^11^ whereas disparities in ${\mathrm{SpO}}_{2}$ are much less common under higher pulsatility scenarios. This may be due to a combination of reduced signal-to-noise ratio at red wavelengths from melanin absorption and an adverse physiological condition that reduces perfusion/pulsatility. Our preliminary work on computational modeling of pulse oximetry indicated that %Mod—the difference in detected light intensity between systolic and diastolic states divided by the diastolic level—exhibits an inverse correlation with melanin content.^12^ This effect is at least partially corroborated by prior modeling studies.^13^^,^^14^ It should also be noted that a recent clinical study demonstrated that the broad spectral bandwidth of pulse oximeter light sources can result in erroneously high ${\mathrm{SpO}}_{2}$ measurements for patients with higher melanin concentration^15^ whereas narrow band sources are not impacted. Previously, researchers had theorized that this difference might occur as a result of melanin’s strong decay in absorption with wavelength. Thus, for strong pigmentation, the epidermis may act as a “variable light filter,” increasing the centroid wavelength of red light-emitting diode (LED) emissions.^16^ Additional research is needed to validate these potential mechanisms.

Healthy human volunteer testing involving controlled desaturation to SaO_2_ levels as low as 70% is recommended to establish the performance of pulse oximeters,^17^^,^^18^ yet this testing is burdensome, expensive, and not without risk for human subjects. With increasing scrutiny of performance disparities, more extensive human subject testing may be needed to ensure equitable performance, exacerbating these challenges. However, this need could potentially be alleviated through the development of realistic benchtop performance test methods capable of predicting real-world performance. In addition, phantom-based tools can facilitate an understanding of light–tissue interactions and factors impacting device performance, leading to the development of novel solutions. Phantoms represent a common method for evaluating the performance of biophotonic techniques,^19^^–^^21^ and their use has been recommended in standards for cerebral oximetry^17^ and functional near-infrared spectroscopy.^22^ Furthermore, phantom-based testing can augment clinical findings by providing insight into device performance outside safe physiological ranges for human volunteer subjects, such as ${\mathrm{SaO}}_{2}<70\%$, high dyshemoglobin levels, or low hematocrit levels. However, there is currently a lack of well-validated tissue-mimicking phantoms for objective, quantitative evaluation of pulse oximeter performance, including robustness to variations in skin pigmentation.

Several researchers have attempted to create tissue-relevant phantoms for pulse oximetry. An early finger phantom comprising acrylic and silicone was used to test accuracy, reproducibility, and linearity.^23^ In subsequent studies, glass and plastic blocks were used to mimic the finger.^24^^–^^27^ However, pulse oximetry phantoms should replicate the mechanical and optical properties of tissues under consideration. By exhibiting biologically realistic mechanical elasticity, a phantom enables the replication of transient cyclical variations in blood volume. This will produce variations in detected optical signals—transmission, in the case of finger sensors—which form the basis of the PPG waveform. The glass and plastic phantoms mimic scattering properties, replicating the optical properties of the tissue. Silicone has been used to simulate the blood vessels because its flexibility enables the generation of PPG signals. Even when optical properties and blood vessel flow velocities are represented, physical phantoms have often lacked the geometry and mechanical properties to accurately simulate human tissue. Researchers have used different materials for the fabrication of phantoms which may be categorized as rigid material-based phantoms and flexible/soft material-based phantoms.

Several promising approaches for fabricating pulse oximetry phantoms have been published in recent years.^28^^–^^30^ Nomoni et al. used a combination of continuous dip coating, three-dimensional (3D) printing, and manual assembly to create polydimethylsiloxane (PDMS)-based tubes with realistic mechanical properties that were incorporated into a vascular tissue phantom. However, this phantom involved a single linear channel and flat sides that are likely incompatible with finger sensors. Research by Gylys et al.^28^ implemented a flow system with adjustable blood oxygenation and a peristaltic pump along with a “pulsatile cuvette” that was not described in detail or material properties provided; thus, the biorelevance of this method is unclear. Jenne and Zappe^29^ developed recipes for PDMS-based phantoms that provide realistic mechanical properties and assessed material compliance with a microscopy approach. They implemented a multi-layer phantom with a single channel and rectangular geometry that would likely be incompatible with common pulse oximeter finger sensors. In a recent study, we investigated the potential for fabricating 3D printed phantoms for pulse oximetry.^30^ Although this approach can generate realistic, readily disseminated phantom morphology and offers flexibility in customizing optical properties, we found that currently available polymers are not sufficiently compliant.^30^ Each of these phantom-based approaches appears inadequate for clinical finger sensor pulse oximeters, and testing on actual devices was not reported.

The purpose of our current research is to streamline the clinical realization of advances in optical technologies that enhance public health—including the mitigation of disparities—through the development of regulatory science tools. Specifically, our goals were to develop a tissue-mimicking material (TMM) with biorelevant physical properties and assess whether a prototype phantom based on this TMM can produce predictable, tunable behavior and outputs, including PPG signals and SpO_2_ values, when measured with pulse oximeters.

## 2 Methods

The initial phase of this research involved the development of composite TMMs. In an iterative process, candidate silicone-based materials were custom-formulated and then characterized using spectrophotometry and two mechanical property measurement methods to achieve target properties. Flow-channel phantoms made from two of these TMMs were evaluated for vessel compliance with optical coherence tomography (OCT). Finger phantoms with channels were created using a hybrid approach where a bulk finger mold and a water-soluble filament were 3D printed and then used to form and cure samples made from each of the TMMs. In addition, this study uses epidermis phantoms developed in our prior project to simulate variations in pigmentation.^31^ Finally, phantoms were connected to a pulsatile fluid system, and PPG waveforms were measured by research and commercial pulse oximetry devices. Details on each of these approaches are provided in this section.

### 2.1 Material Preparation

Polymer composites such as silicone and PDMS are commonly used to create phantoms^32^^–^^36^ for evaluating and calibrating optical systems. PDMS has been implemented in biophotonics research as a TMM^29^^,^^31^^,^^37^ due to its flexibility, non-toxicity,^38^ and ease of mechanical property tuning.^39^ In this work, we tested a well-known PDMS product (SYLGARD™ 184, Dow, Midland, Michigan, United States) as well as a more novel platinum-catalyzed silicone (Ecoflex™, Smooth-On, Inc., Macungie, Pennsylvania, United States) that is gaining popularity due to its tissue-relevant mechanical properties—particularly a low Young’s modulus—and ease of handling.^40^^,^^41^

Target hardness values were chosen as 23.4 to 31.8 Shore OO based on measurements of all fingertips from 10 volunteers,^42^ whereas target elastic modulus values were chosen as 34 to 136 kPa, which covers the range of elastic modulus of the subcutaneous tissue, dermis, and epidermis.^43^ The manufacturer guidelines for PDMS recommend a base-to-curing-agent ratio of 10:1 and curing conditions of either 150°C for 10 min, 125°C for 20 min, 100°C for 45 min, or room temperature for 48 h.^44^ Initially, PDMS samples were formulated with the recommended curing agent concentration of 10% by weight at room temperature for 48 h. We studied the effect of reducing curing agent concentration as low as 3% to reduce PDMS rigidity.^38^^,^^45^^,^^46^

Three Ecoflex products with different levels of rigidity—00-20, 00-30, and 00-50 (corresponding to nominal Shore OO hardness of 20, 30, and 50)—were fabricated by mixing equal parts (parts A and B) by weight and curing at room temperature. To evaluate fine-tuning of mechanical properties, equal part mixtures of 00-20 and 00-30 as well as 00-30 and 00-50 were formulated (labeled as “Mix 20-30” and “Mix 30-50”).

TMM preparation involves manual mixing, which generates air bubbles in the solution. The solution was then degassed in a 5-gallon chamber connected to a vacuum pump (Model 003-PT-30172 1-Stage Vacuum Pump, Arksen, City of Industry, California, United States). Degassing was performed until bubbles were removed, which required 20 min in PDMS and 2 min in Ecoflex. All samples were prepared and poured in the master mold within the pot life period of TMMs—2 h for PDMS and 15 to 30 min for Ecoflex at room temperature.

### 2.2 Mechanical Characterization—Shore Hardness and Elastic Modulus

Elastic modulus and Shore hardness have been used to characterize TMM mechanical properties for the development of phantoms.^30^^,^^47^^,^^48^ Although the former represents a common approach for mechanical property characterization, the latter provides a more convenient albeit less rigorous measurement. In this study, we collected data using both approaches, in part to assess the viability of using hardness in future studies.

**Fig. 1:**
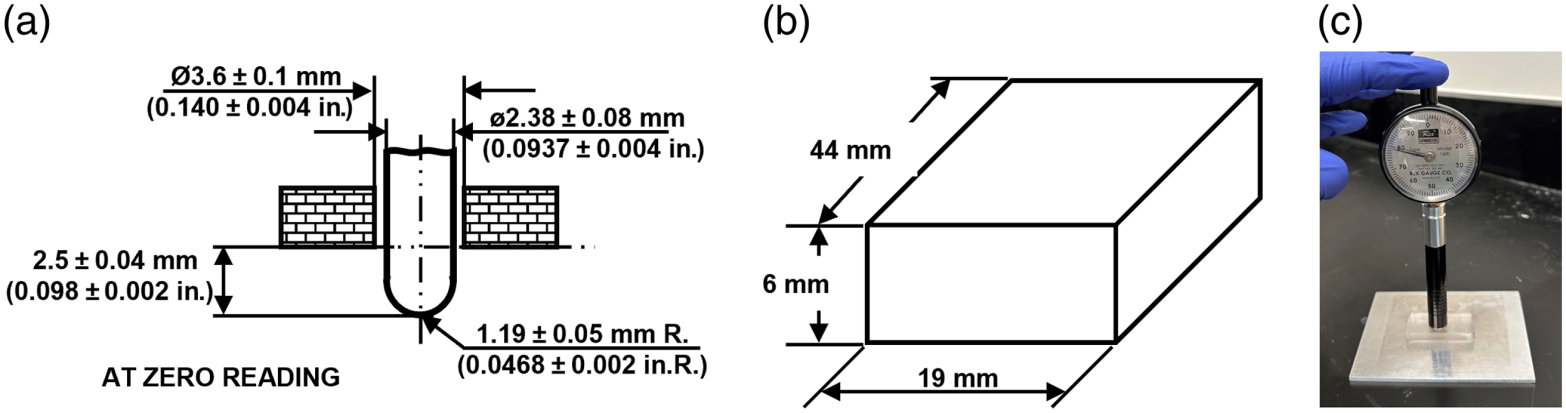
Shore hardness (Shore OO) measurement of material. (a) Schematic type OO indentor of durometer.^49^ (b) Schematics of the sample prepared for hardness testing. (c) Image of the sample measured using a durometer.

Shore hardness is measured using a durometer, which quantifies indentation resistance. We followed a standard for hardness measurement in rubber, ASTM D2240–15,^49^ and used a Shore hardness OO scale durometer (Model 1600-OO Dial Durometer, Rex Gauge LLC, Buffalo Grove, Illinois, United States) that incorporates a spherical indentor and analog dial display [Fig. 1(a)]. TMM rectangular block samples (44×19×6  mm) were molded [Figs. 1(b) and 1(c)], and three readings were acquired using the durometer placed vertically over the sample surface at different locations.

**Fig. 2:**
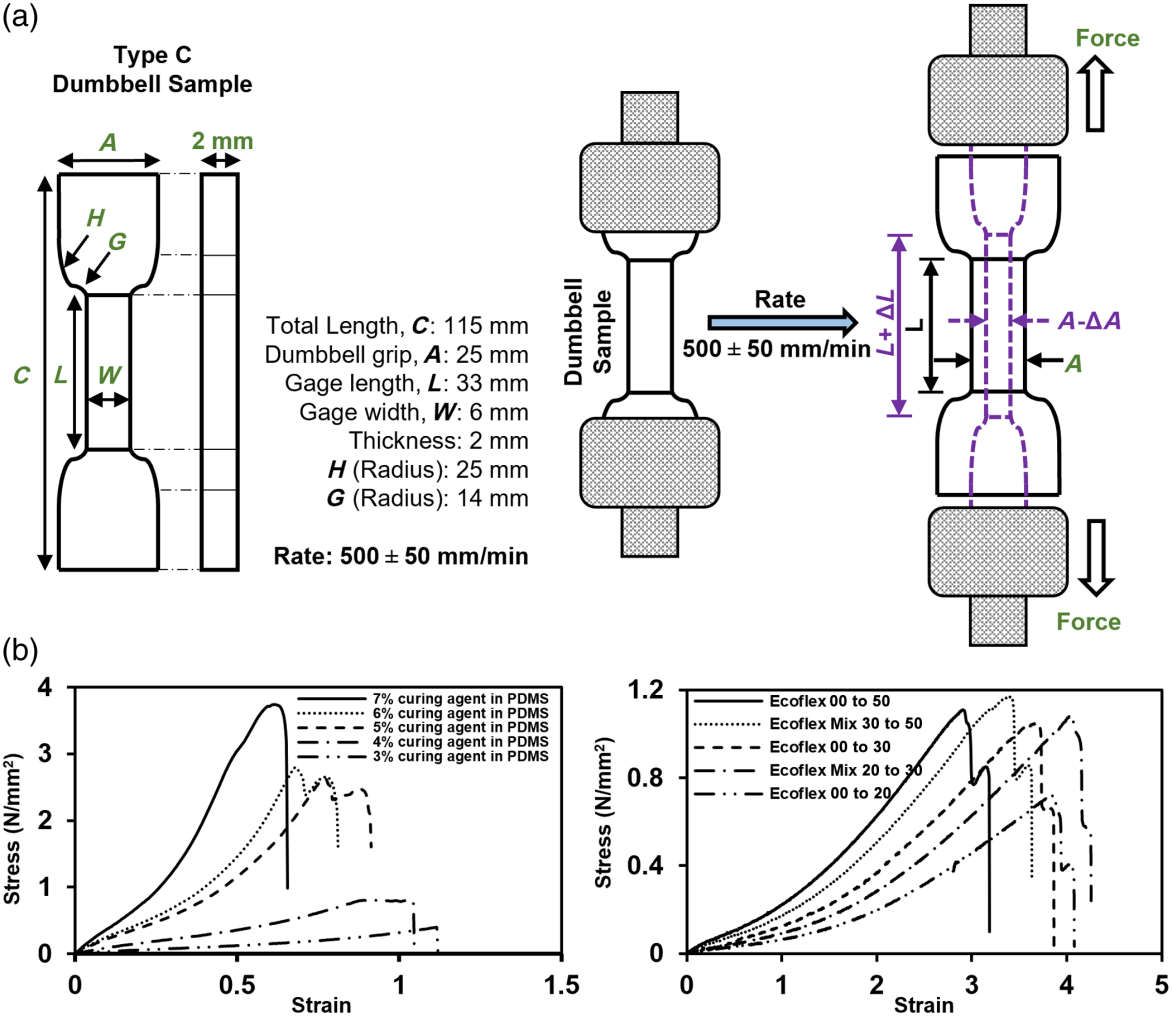
(a) Setup for tensile testing to measure elastic modulus of silicone samples. (b) Example of stress–strain curve of different samples of PDMS (left) and Ecoflex (right).

Elastic moduli were determined using a tensile test according to ASTM D412(C),^50^ which prescribes a dumbbell-shaped specimen geometry [Fig. 2(a)] in test method “C” of the standard.^50^ To fabricate a dumbbell-shaped specimen, a rectangular sample of 2 mm thickness was molded, and a dumbbell shape was created using an ASTM D-412(C) cutting die. PDMS and Ecoflex specimens were then placed in a universal testing machine (33R-4465, Instron^®^, Norwood, Massachusetts, United States), where samples were placed in tension at a constant strain rate of 500±50  mm/min, as specified by the ASTM standard, whereas force and displacement values were recorded. Stress (σ) and strain (ε) values [Fig. 2(b)] were then calculated from force and displacement. Elastic modulus was calculated from the slope of the stress–strain curve via Hooke’s law as E=σ/ε. Because the stress–strain curve is generally non-linear for elastomers, we chose to calculate elastic modulus by linear regression over lower strains (0≤∈≤0.5) where the curve is more linear.

### 2.3 Mechanical Characterization—Compliance

The ability of a vessel lumen to expand as a function of pressure within the lumen is referred to as compliance and is typically defined as the ratio of change in cross-sectional area to the change in pressure (δs/δp).^51^^,^^52^ The phantom channel should produce biologically realistic volumetric expansion when the internal pressure is increased over a biorelevant range (typically 80 to 120 mmHg).^53^ Rather than using traditional methods,^29^^,^^54^^,^^55^ we developed a novel approach for non-invasively measuring phantom channel diameter changes during pulsatile flow using OCT. A research-grade, spectral-domain OCT system^56^ was used with a central wavelength of 1070 nm and 86 nm bandwidth, providing a lateral resolution of 16  μm and an axial resolution of 12  μm. The system images samples through a 1.6× magnification telecentric objective lens. Phantom channels were imaged in cross-section with an image size of 550 pixels (5 mm) in the lateral direction and 1024 pixels in the axial direction. Axial pixel size was determined to be 5.6  μm in air ($z_{\text{air}}$) and validated using a 1-mm microscope glass slide with a refractive index of 1.5. Axial pixel size in the channel was calculated as $z_{\text{channel}}=z_{\text{air}}/n_{c}$, where $n_{c}$ is the refractive index of the deionized (DI) water in the channel ($n_{c}=1.33$). In addition, 20 B-scans were collected at the same location at 125 fps and averaged before measurements. Channel walls were identified by locating peaks in the OCT image and processed to quantify channel dimensions.

**Fig. 3:**
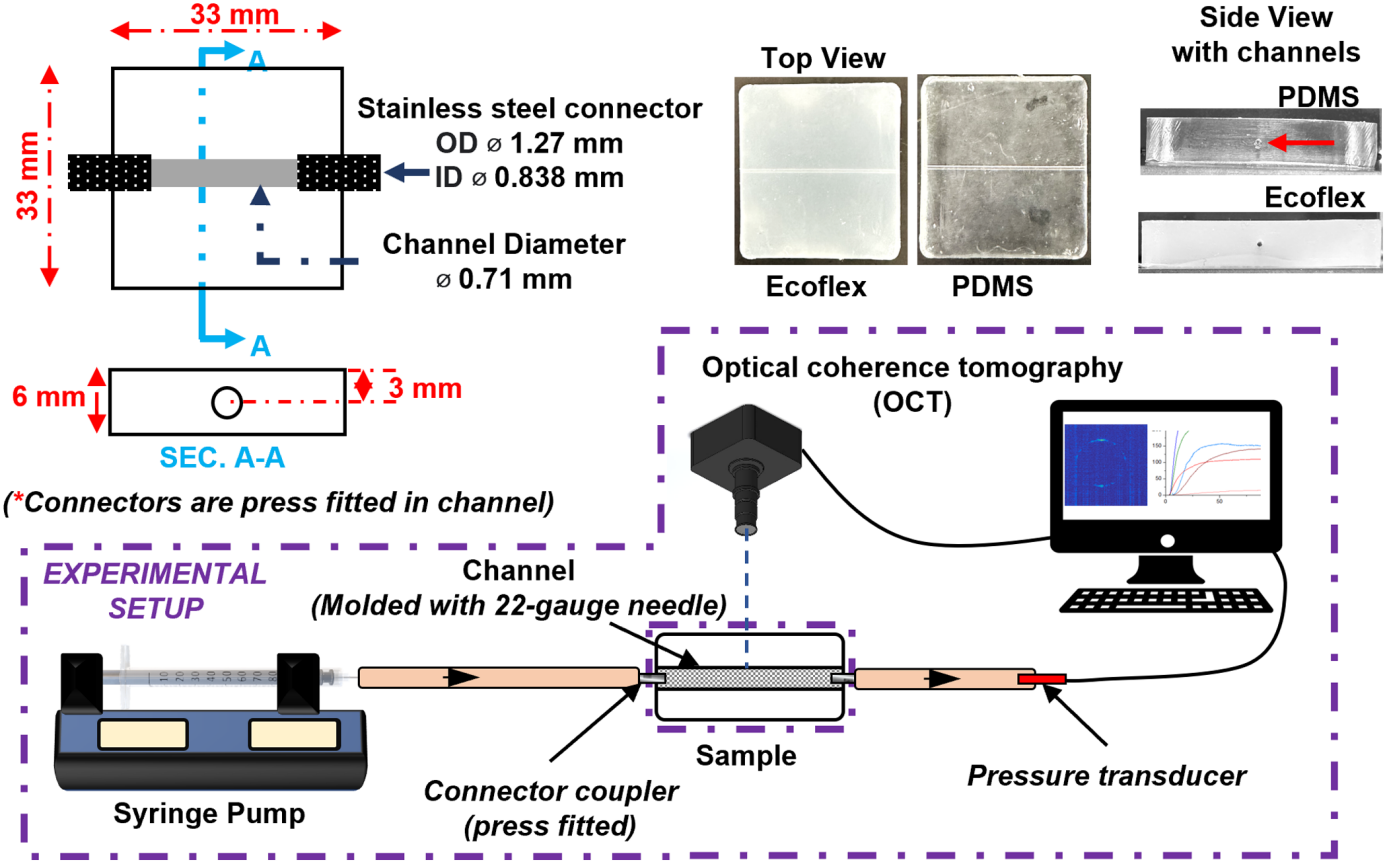
Compliance test: technical drawing of samples prepared for compliance test (top left), images of Ecoflex and PDMS samples with channel (top right), and schematics of the experimental setup for compliance test to measure the change in the diameter of the channel as a function of change in pressure (bottom).

A simple rectangular block phantom (33×33×6  mm) was fabricated with a 0.71-mm diameter channel by inserting a 22-gauge needle through the phantom material at a depth of 3 mm before curing. Ecoflex samples were cured for 4 h at room temperature, whereas PDMS was cured at 150°C for 2 h. The channel was connected using a needle coupler and tubes to a positive displacement syringe pump (Model LEGATO 270, KD Scientific Inc., Holliston, Massachusetts, United States) on one side and a pressure measurement system on the other (Fig. 3). The increase in pressure was achieved by gradual displacement of the plunger in a syringe. Polyvinyl chloride tubing (Tygon®, Saint-Gobain Corporation, Courbevoie, France) with Shore 60A was selected because its higher hardness relative to the TMMs ensures negligible impact on lumen volume change. The pressure measurement system consisted of a pressure recording unit (Millar PCU-2000, Millar, Houston, Texas, United States) and catheters (MPR-500 Mikro-tip pressure catheter, Millar, Houston, Texas, United States), with data acquired at 1 kHz. To characterize compliance, pressure in the channel was increased gradually from 0 to 180 mmHg using the syringe pump as OCT images were acquired. Finally, the compliance was determined as the ratio of change in cross-sectional area to the change in pressure.

### 2.4 Optical Characterization

Careful consideration of tissue optical properties is important for designing a phantom intended to simulate pulse oximeter light-tissue interactions. TMMs based on Ecoflex and PDMS were customized to target literature values for $\mu_{a}$ and $\mu_{s}^{\prime }$ of the human finger from the literature. Rodriguez et al.^30^ reported bulk optical property averages for the index finger at 660 and 940 nm: $\mu_{a}$ was 0.9 and $1.1\;{\mathrm{cm}}^{-1}$, and $\mu_{s}^{\prime }$ was 9.8 and $14.7\;{\mathrm{cm}}^{-1}$, respectively. As blood is the dominant chromophore in sub-epidermal regions of the finger, we have attempted to match volumetric blood content in the phantom channels^57^ but have not added other background absorbers. This approach is similar to that used in our cerebral oximetry phantom study.^31^ To adjust $\mu_{s}^{\prime }$, we doped the base material with 0.75 to 1.25 mg/g titanium dioxide (${\mathrm{TiO}}_{2}$) (CAS No: 1317-70-0, Sigma-Aldrich, St. Louis, Missouri, United States). For Ecoflex, we mixed the desired amount of ${\mathrm{TiO}}_{2}$ in 400  μL ethanol by bath sonicating for 30 min at room temperature, added 15 g of Ecoflex (7.5 g of part A: 7.5g of part B), and degassed and cured for 4 h at room temperature. The solution was then poured into a ring-type mold of 40 mm diameter and 2 mm thickness. The mold was positioned between two aluminum plates, and the TMM was crosslinked to achieve circular disc samples. Optical properties were characterized using a dual-beam integrating sphere spectrophotometer (Lambda 1050, PerkinElmer, Waltham, Massachusetts, United States). Total transmittance and diffuse reflectance spectra were acquired from a sample, and $\mu_{a}$ and $\mu_{s}^{\prime }$ were calculated using the inverse adding-doubling (IAD) algorithm,^58^ assuming an anisotropy factor of 0.9 and a refractive index of 1.4. The consistency of IAD results was verified by taking measurements of an Ecoflex 00 to 30 sample with 1.25 mg/g ${\mathrm{TiO}}_{2}$. Ten readings were acquired, and $\mu_{a}$ and $\mu_{s}^{\prime }$ were measured. The coefficient of variation was found to be <10% for both properties (Fig. S1 in the Supplementary Material). Although our phantoms do not contain added bulk absorbers, the addition of blood in the channels and melanin in the epidermal layer provide the dominant tissue chromophores. To achieve biorealistic bulk absorption based on the literature,^59^^,^^60^ we have incorporated channels (described below) that provide a blood volume fraction of ∼1.135%. When we implement the final version of our phantom, we will re-evaluate this fraction and, if necessary, add a bulk absorber to ensure clinically relevant signals.

### 2.5 Phantom Fabrication

**Fig. 4:**
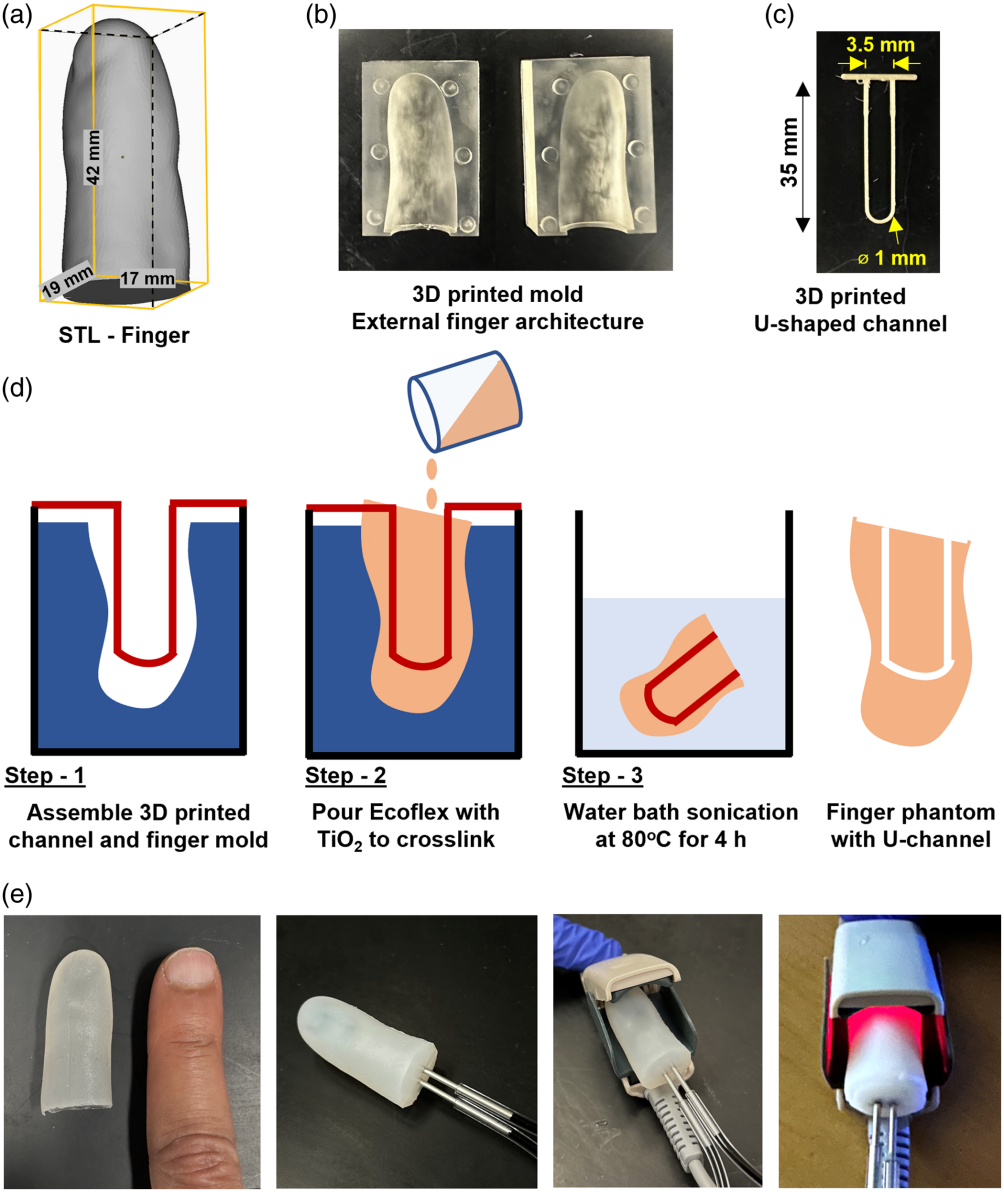
Pulse oximetry phantom fabrication. (a) CAD file of an adult finger. (b) 3D printed phantom mold. (c) 3D printed channel filament. (d) Illustration of the Ecoflex phantom molding. (e) Images of a completed phantom, attached to feeder tubes and placed in the pulse oximeter for PPG measurements.

Final finger-mimicking phantoms were fabricated with a hybrid approach involving 3D printing and molding. A stereolithography 3D printer (Form 3, Formlabs, Somerville, Massachusetts, United States) was used to print the finger mold with high-temp resin (FLHTAM02, Formlabs, Somerville, Massachusetts, United States). An extrusion printer (i3 MK3S+, Prusa, Prague, Czech Republic) was used to print the vessel-forming filament from a proprietary water-soluble material (PrimaSelect PVA+, Prusa), the filament acts as a sacrificial dissolvable material to create internal channels in finger phantom (Fig. S2 in the Supplementary Material). To create physiologically relevant finger phantoms, a stereolithography (STL) model of the distal phalanx [Fig. 4(a)] was extracted from a digital model of the hand^61^ and used to print a mold [Fig. 4(b)] that forms the external phantom surface. A digital model of the U-shaped vessel-forming filament (1 mm in diameter and 35 mm in length from the tip to the trough of the U shape) was generated and printed [Fig. 4(c)]. The filament was inserted into the finger mold and uncured liquid TMM was poured and crosslinked for 4 h at room temperature. The crosslinked finger phantom was then extracted from the mold with the embedded filament and sonicated in a water bath at 80°C for up to 4 h. There was no change in the hardness of the material with additional heating at 80°C up to 4 h (Table S1 in the Supplementary Material). During the sonication process, the phantom was flushed every 30 min with DI water from the bath in a syringe until the channel was completely cleared. Figure 4(e) shows a finger phantom prototype made from Ecoflex 00 to 30. To clean the phantom and tubing after an experiment, we used DI water, rinsing the entire setup multiple times. This process was sufficient to clean the tube and the phantom so that no staining was visible. For pulse oximeter measurements, we used epidermis-simulating phantom layers developed in our prior study to simulate melanosome volume fractions ($M_{f}$) of 0%, 6%, 14%, 30%, and 43%.^31^ A list of materials used to fabricate these phantoms is provided in Table S2 in the Supplementary Material.

### 2.6 Pulsatile Fluid Testing of Pulse Oximeter Phantoms

**Fig. 5:**
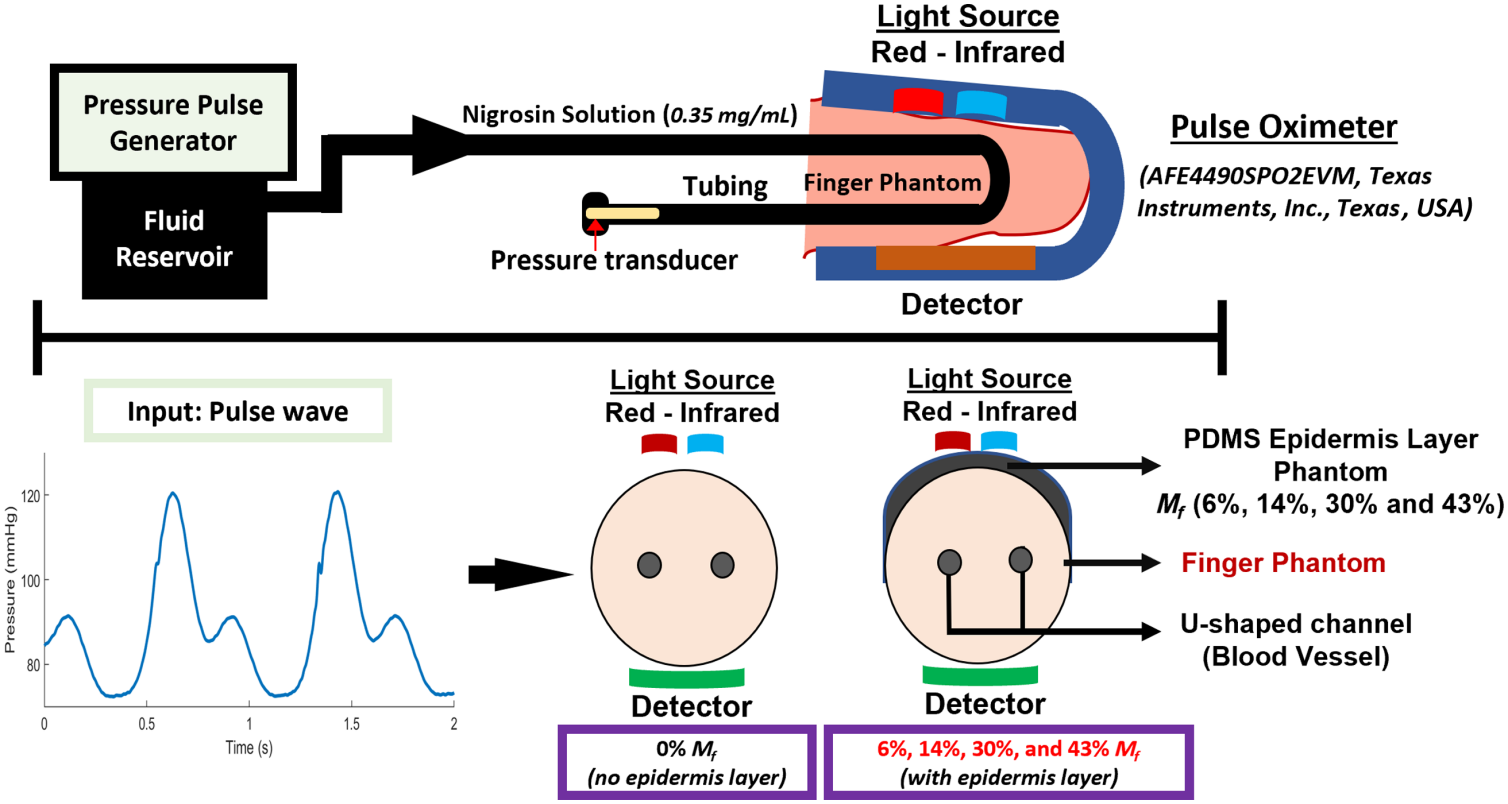
Experimental flow system setup to measure the PPG signal in finger phantom using a pulse oximeter: (top) overall system schematic, (bottom left) input pulse wave, and (bottom right) cross-sectional schematic of phantoms without and with pigmented epidermal layer.

Our final set of measurements involved the implementation of the Ecoflex finger phantom in a pulsatile fluid system with a pulse oximeter as a preliminary validation of the phantom’s ability to generate PPG signals over a wide, clinically relevant range of %Mod levels. The closed-end fluid system (Fig. 5) is designed using a pressure pulse generator (PPG-601A, Flometrics Inc., Carlsbad, California, United States) capable of generating a wide range of pressures (0 to 200 mmHg). The generator was connected to the finger phantom with rigid tubing. PPG measurements were performed with a pulse oximeter evaluation module (AFE4490SPO2EVM, Texas Instruments, Inc., Dallas, Texas, United States) that recorded PPG waveform outputs at 655 and 900 nm. Pressure signals were recorded as shown in the schematic diagram of the setup (Fig. 5). A complete list of instruments used in the pulsatile fluid system is provided in Table S3 in the Supplementary Material. To approximate blood absorption, different concentrations of water-soluble nigrosin (0.1 to 1 mg/mL) in DI water were tested [Fig. S3(a) in the Supplementary Material]. Our intent was not to simulate a specific ${\mathrm{SpO}}_{2}$ level but rather to provide a blood-like absorption level so that a pulse oximeter under test can output biologically relevant PPG waveforms. A concentration of 0.35 mg/mL yielded $\mu_{a}$ values of 13.9 and $4.0\;{\mathrm{cm}}^{-1}$ at 660 and 940 nm, respectively [Fig. S3(b) in the Supplementary Material], which represents moderately saturated blood.^57^

One key goal of the PPG measurements was to assess the system’s ability to achieve a biorelevant range of %Mod values. %Mod normally ranges from 0.02% to 20%—for very weak to very strong pulses,^62^^,^^63^ and recent clinical evidence indicates that low %Mod levels (<1%) greatly increase the potential for pigmentation-dependent bias.^11^^,^^64^

We simulated two different scenarios using the in vitro pulsatile fluid system: (a) a constant diastolic pressure with varying systolic pressure, thus effectively changing the pulse pressure differential (Table 1) to assess the ability to achieve different %Mod levels, and (b) a constant diastolic and systolic pressure (80/120 mmHg) with changing $M_{f}$ from 0% to 43%. In both scenarios, we maintained the pulse rate at 75 beats per minute (BPM) and applied 0.1-mm-thick epidermal phantom layers developed in our prior project.^31^ The pigmented layer was placed between the light source and the finger phantom to mimic the real-world scenario where the light source is located on the dorsal side of the finger slightly proximal to the fingernail. This location tends to be a melanin-rich region whereas the palmar side contains much less melanin. Although this arrangement represents a slight misplacement of the sensor from its ideal location over the fingernail, it provides a simplified maximum absorption case that is clinically relevant.

**Table 1:** Systolic and diastolic pressures and resulting pulse pressure (systolic minus diastolic), used to generate input pressure waveform for flow phantom testing.

	Pressure 1	Pressure 2	Pressure 3	Pressure 4	Pressure 5	Pressure 6	Pressure 7	Pressure 8	Pressure 9
Systolic peak pressure (mmHg)	65	70	80	90	100	120	140	160	180
Diastolic pressure (mmHg)	60	60	60	60	60	60	60	60	60
Pulse pressure (systolic — diastolic) (mmHg)	5	10	20	30	40	60	80	100	120

Validation of the basic functionality of the finger phantom setup was achieved through measurements with two pulse oximeters (Model Onyx Vantage 9590, Nonin, Plymouth, Minnesota, United States, and Mighty Sat, Masimo, Irvine, California, United States). Although the aforementioned preliminary phantom testing in a research pulse oximeter was performed using nigrosin for testing in clinical pulse oximeters, we implemented India ink (Speedball). The $\mu_{a}$ spectrum simulated a saturation level that was more in line with the range over which pulse oximeters are used clinically. With a concentration of 1.8  μL/mL, the perfused solution exhibited $\mu_{a}$ values of 7.6 and $5.5\;{\mathrm{cm}}^{-1}$ at 660 and 940 nm, respectively (Fig. S4 in the Supplementary Material). This approximated blood at ${\mathrm{SaO}}_{2}=70\%$.^57^

## 3 Results

### 3.1 Mechanical Properties—Shore Hardness

**Fig. 6:**
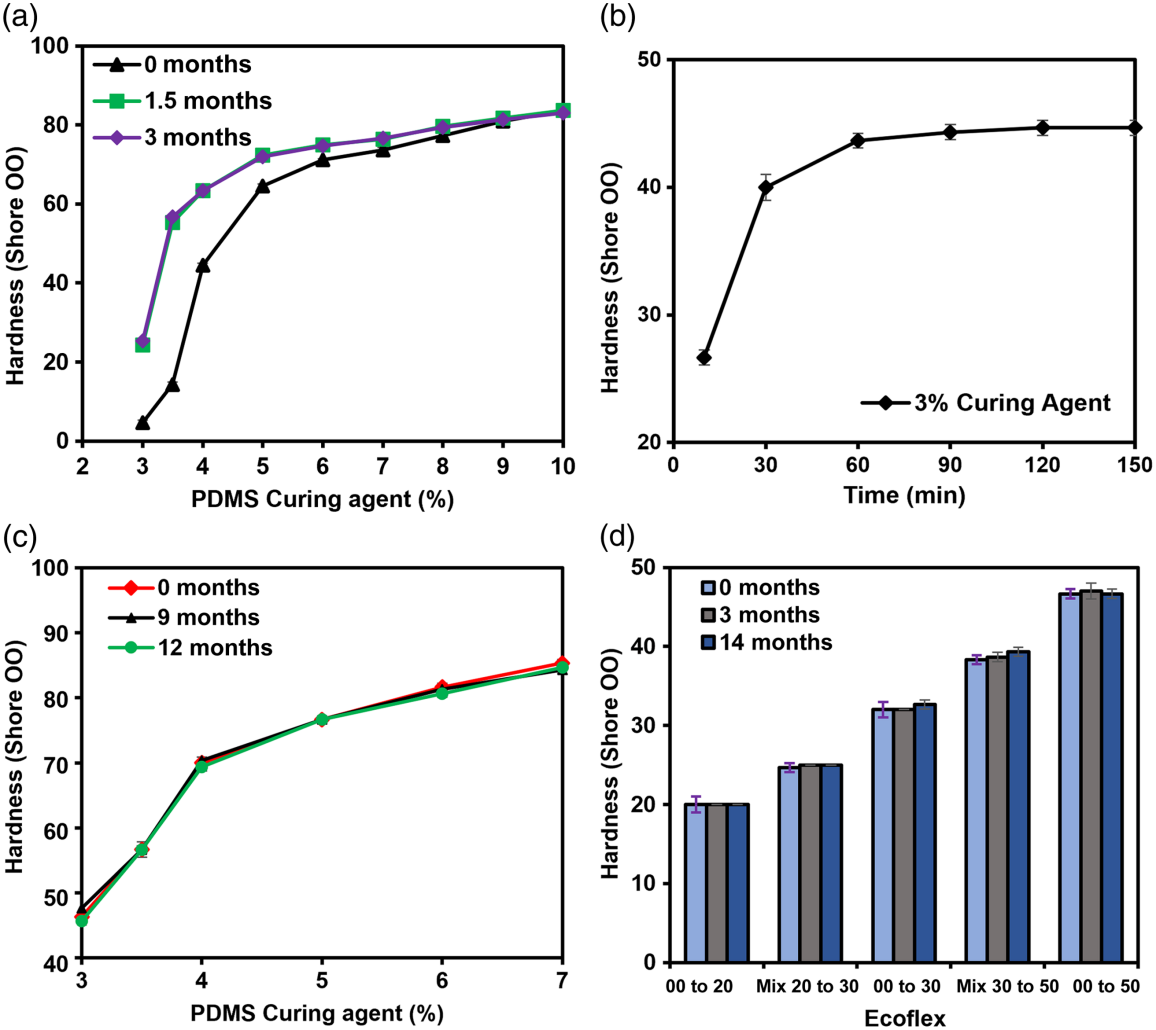
Candidate TMM mechanical property results. (a) Hardness as a function of curing agent level at different post-fabrication time points cured at room temperature for 48 h. (b) Hardness (3% curing agent) as a function of cure duration when heated to 150°C. (c) Hardness of PDMS as a function of curing agent concentration up to 12 months, when cured at 150°C for 2 h. (d) Hardness of different Ecoflex formulations cured at room temperature for up to 14 months.

When implementing the manufacturer-recommended fabrication protocol (10:1 ratio of base to curing agent), PDMS Shore OO hardness was measured as 83.7±0.6, which greatly exceeded the target value. Hardness was observed to decrease with reduced curing agent concentration, with more rapid reduction observed at curing agent concentrations below 5% [Fig. 6(a)]. PDMS at 3.5% and 4% curing agent concentration exhibited biorelevant hardness values^42^ 14.3±0.6 and 44.5±0.5, respectively. After 1.5 months, PDMS samples showed increased hardness (e.g., 55.3±0.6 versus 14.3±0.6 at 3.5% concentration), indicating that crosslinking was active even after 48 h of curing. After 3 months, minimal additional change (<5%) was noted [Fig. 6(a)]. Samples with lower curing agent concentration exhibited unstable hardness values. To identify an optimum curing protocol, we implemented the highest manufacturer-recommended curing temperature (150°C) cured for durations up to 2 h [Fig. 6(b)]. PDMS hardness increased with a curing duration of up to 60 min, whereas higher durations produced only marginal increases in hardness (<2.5%). Hardness was stable within 3% after 12 months of fabrication, for all concentrations [Fig. 6(c)]. Thus, for subsequent fabrication, we focused on curing PDMS samples at 150°C for 2 h. By reducing PDMS curing agent concentrations from 7% to 3%, we observed a gradual reduction in hardness from 85.3±0.6 to 46.3±0.6 [Fig. 6(c)]. However, the lowest hardness value of 46 is greater than the target value, and the curing agent concentration could not be further reduced due to difficulties in handling (e.g., stickiness and breakage). Therefore, additional testing of PDMS samples in this study focused on 3% curing agent concentration.

Hardness increased linearly with increasing amount of stiffer Ecoflex products used in formulation, with values for Mix 20 to 30 and Mix 30 to 50 consistent with the average hardness of their respective components (within 4%) [Fig. 6(d)]. Ecoflex Mix 20 to 30 and 00 to 30 had hardness values in the range of target values 42. Samples of 00 to 20, Mix 20 to 30, and 00 to 30 showed <1% change in hardness up to 14 months after fabrication [Fig. 6(d)].

### 3.2 Mechanical Properties—Elastic Modulus

**Fig. 7:**
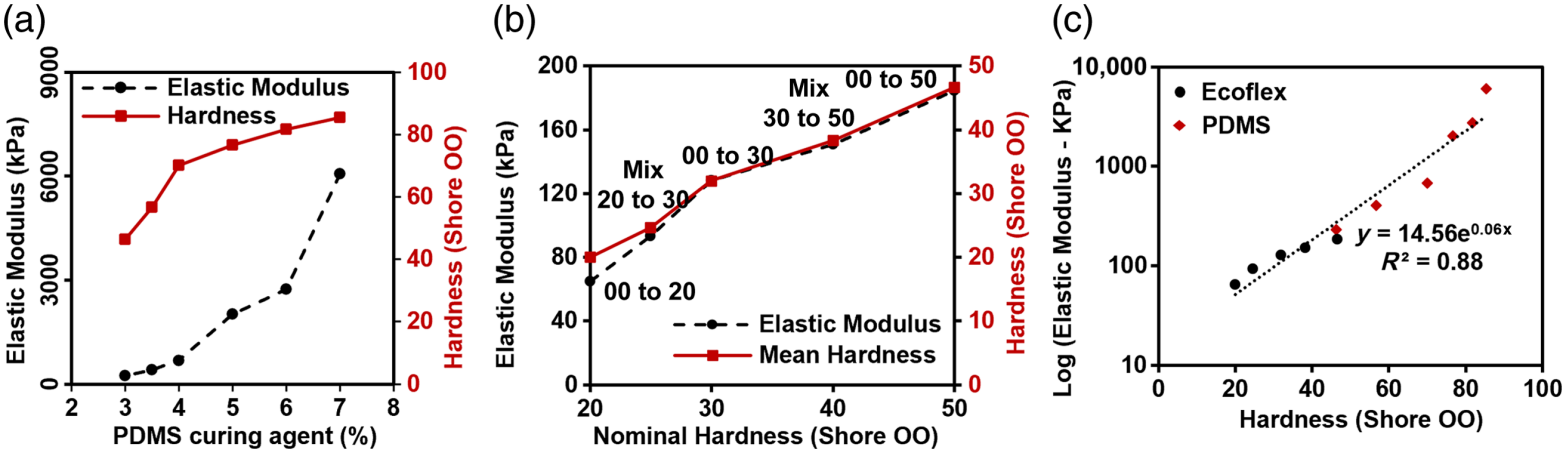
Mechanical property results. Elastic modulus and hardness of (a) PDMS at different curing agent concentrations. (b) Elastic modulus and hardness of Ecoflex formulations. (c) Log (elastic modulus) versus hardness of Ecoflex (circles) and PDMS (diamonds).

Measurements of elastic modulus in PDMS and Ecoflex samples provide insight into the tunability of mechanical properties as well as the viability of simpler measurements of hardness. PDMS samples were prepared by mixing the curing agent and base PDMS and cured at 150°C for 2 h. Although PDMS hardness increased with diminishing returns with increasing curing agent concentration above 4%, PDMS elastic modulus exhibited more substantial increases at higher curing agent concentrations [Fig. 7(a)]. For Ecoflex formulations with nominal Shore OO hardness values of 20 to 50 (i.e., Ecoflex 00 to 20 and 00 to 50) [Fig. 7(b)], elastic modulus values ranged from 65 to 184 kPa. As with hardness values, binary mixtures of two Ecoflex materials exhibited an elastic modulus close to the mean of the two individual components. Both curves in Fig. 7(b) exhibit similar trends, with decreasing slopes at higher nominal hardness levels.

A comparison of elastic modulus and hardness was performed to assess whether a simple conversion exists among these metrics. Results in Fig. 7(c) indicate an approximately exponential relationship between these two parameters. Using the fitting equation displayed in this graph ($E=14.56\times 10^{0.6*\mathrm{hardness}}$), converting between hardness and elastic modulus can be readily achieved $\left(\mathrm{hardness}=\frac{\log(E/14.56)}{0.06}\right)$. Thus, a relatively simple durometer measurement may be suitable *in lieu* of more complex and expensive tensile testing for these materials.

### 3.3 Mechanical Properties—Compliance

**Fig. 8:**
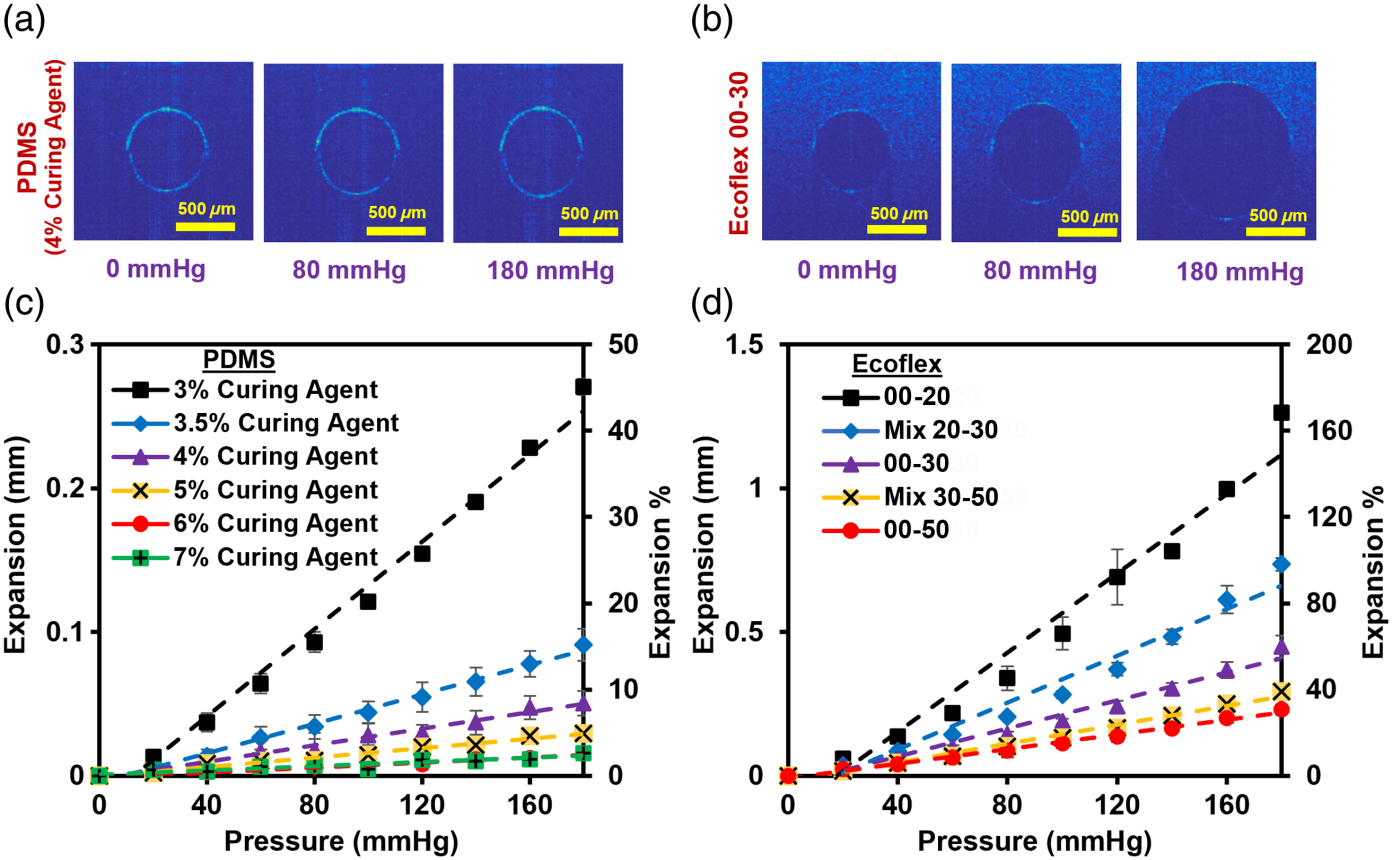
Expansion of channels (increase from initial diameter of 0.71 mm) as a function of pressure. (a) OCT images of PDMS (4% curing agent) at 0, 80, and 180 mmHg. (b) OCT images of Ecoflex 00 to 30 at 0, 80, and 180 mmHg. (c) PDMS expansion versus pressure. (d) Ecoflex expansion versus pressure.

Materials with biologically relevant mechanical properties should enable significant changes in volume when physiologically relevant pressure levels are applied in phantom channels. Results of compliance tests [Figs. 8(a) and 8(b)] include OCT images of channels in PDMS with 4% curing agent and Ecoflex 00 to 30 (with Shore OO hardness of 70 and 32, respectively). These data illustrate how increasing pressure (0, 80, and 180mm Hg) expands the channels. Channel diameters increased by up to 184% and 40% for the softest Ecoflex and PDMS materials, respectively [Figs. 8(c) and 8(d)]. Data in Figs. 8(c) and 8(d) also illustrate the inverse relationship between hardness and degree of expansion (diameter). For example, at 180 mmHg in PDMS with a 7% curing agent, the level of expansion was 2.3%, whereas for PDMS with a 3% curing agent, 39% expansion was seen [Fig. 8(c)]. A similar effect was observed for Ecoflex, where types 00 to 50 and 00 to 20 produced expansion of up to 33% and 185%, respectively [Fig. 8(d)]. The study shows that a range of expansion from 2.3% to 184.5% at a pressure of 180 mmHg may be achieved by tuning Ecoflex and PDMS compositions. In addition, Table 2 shows the percentage expansion of straight channels in all the materials tested as a function of biorelevant pressure of 80 to 120 mmHg.

**Table 2:** Radial expansion of 0.71-mm-diameter straight channels during a pressure rise of 80 to 120 mmHg in candidate materials.

	PDMS curing agent						Ecoflex				
	3%	3.5%	4%	5%	6%	7%	00-20	Mix 20-30	00-30	Mix 30-50	00-50
Material Expansion %	7.84	2.82	1.72	1.32	0.26	0.53	34.48	18.50	11.46	8.07	6.32

**Fig. 9:**
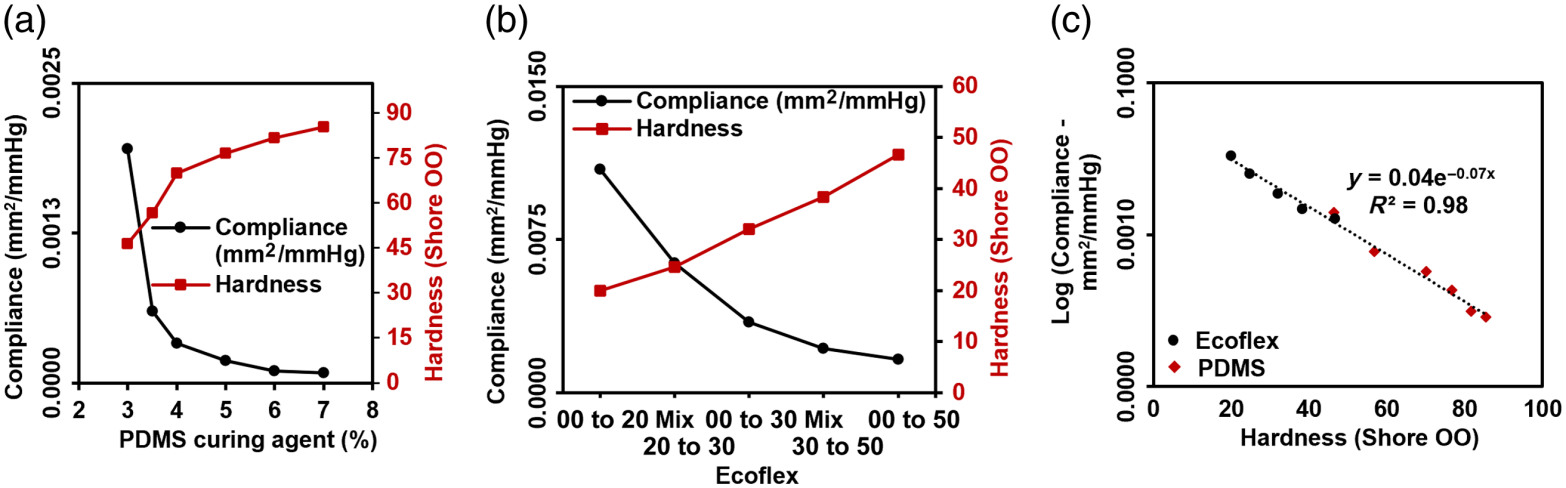
Compliance test result. (a) Compliance and hardness of PDMS as a function of curing agent content. (b) Compliance and hardness of Ecoflex at different combinations. (c) Overall relationship between compliance and hardness for Ecoflex (circular) and PDMS (diamond) samples.

An inverse non-linear relationship was observed between compliance and hardness, with compliance changing greatly across lower hardness levels. Log-scaled data for these two materials overlap to form a nearly continuous relationship [Fig. 9(c)], with the hardest Ecoflex material and the softest PDMS material exhibiting similar hardness and compliance values. This information may be useful for material selection and phantom customization for pulse oximetry and PPG studies.

### 3.4 Optical Properties

**Fig. 10:**
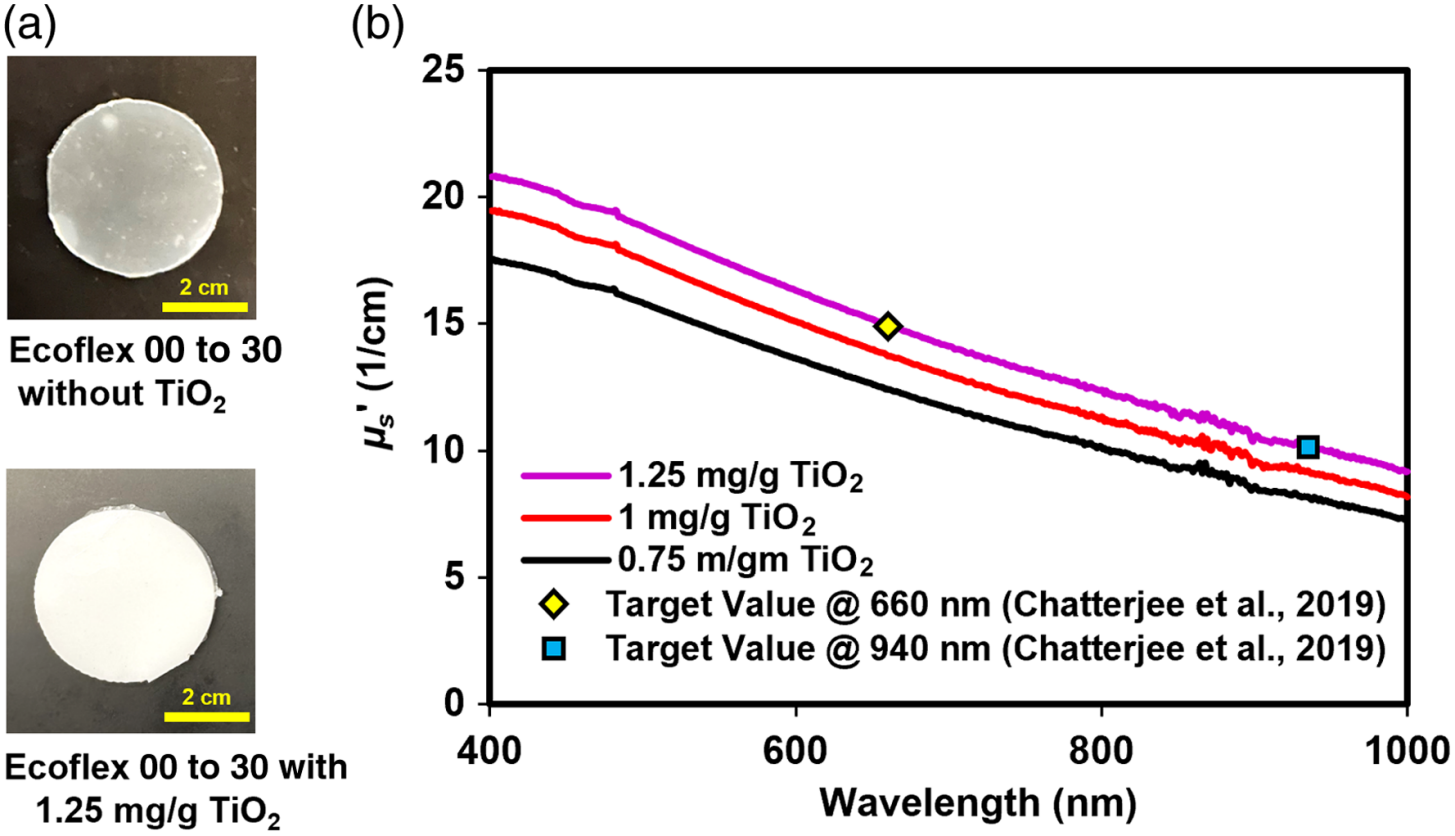
Optical properties of silicone-based TMMs. (a) Photos of Ecoflex 00 to 30 samples with and without TiO_2_. (b) Reduced scattering coefficients versus ${\mathrm{TiO}}_{2}$ concentration.

Candidate TMMs with suitable mechanical properties (Ecoflex 00 to 30, Fig. 10, and PDMS with 4% curing agent, Fig. S5 in the Supplementary Material) were modified through the addition of ${\mathrm{TiO}}_{2}$ particles to provide biologically relevant scattering. Figure 10(a) shows Ecoflex samples with and without scattering prepared for spectrophotometer measurements. Figure 10(b) demonstrates the reduced scattering coefficient ($\mu_{s}^{\prime }$) of Ecoflex 00 to 30 samples doped with ${\mathrm{TiO}}_{2}$ at 0.75, 1, and 1.25 mg/g. As expected, increasing ${\mathrm{TiO}}_{2}$ concentration increased $\mu_{s}^{\prime }$ at all wavelengths, producing a monotonically decreasing scattering coefficient with increasing wavelength, approximating biological tissue. Ecoflex 00 to 30 with 1.25 mg/g ${\mathrm{TiO}}_{2}$ yielded $\mu_{s}^{\prime }$ values consistent with target values^65^ and was selected for finger phantom development. Although the materials provide a small background level of absorption (Fig. S6 in the Supplementary Material) for example, $\mu_{a}$ for Ecoflex 00 to 30 with 1.25 mg/g ${\mathrm{TiO}}_{2}$ was 0.098 and $0.12\;{\mathrm{cm}}^{-1}$ at 660 and 940 nm, which mirrors the impact of minor visible to near-infrared (Vis-NIR) chromophores such as collagen and water, in their final form the blood-filled channels in these phantoms will provide biorelevant absorption levels.

### 3.5 PPG Waveforms

**Fig. 11:**
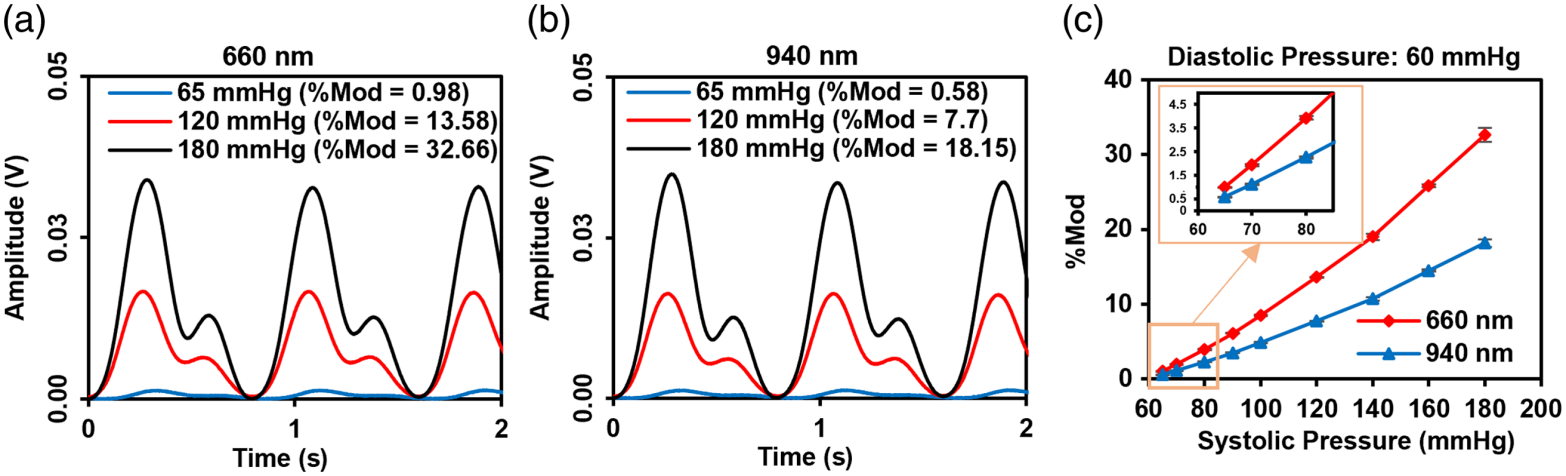
(a) PPG signal waveform at 660 nm for constant diastolic pressure of 60 mmHg and varying systolic peak pressure (65, 120, and 180 mmHg). (b) PPG signal waveform at 940 nm for constant diastolic pressure of 60 mmHg and varying systolic peak pressure (65, 120, and 180 mmHg). (c) %Mod for constant diastolic pressure of 60 mmHg and variable systolic pressure (65, 70, 80, 90, 100, 120, 140, 160, and 180 mmHg).

The finger phantom and flow system described in Secs. 2.5 and 2.6 were used to generate preliminary results to assess the capability of our test method. We attempted to generate biorelevant PPG signals across a range of %Mod values. The ability to adjust %Mod values in a test method is particularly important given recent clinical results indicating that pigmentation-correlated bias in pulse oximeters tends to occur in low %Mod scenarios (e.g., poorly perfused/cold hands).^11^ To achieve a wide range of %Mod values in the measured PPG signals, we varied the input waveform magnitude such that the maximum (systolic) pressure ranged from 65 to 180 mmHg while holding the minimum (diastolic) pressure constant at 60 mmHg. The pulse oximeter measured waveforms at 660 and 940 nm with varying levels of AC amplitude, matching the trends published in prior clinical studies [Figs. 11(a) and 11(b)].^66^ Values of %Mod ranged from 0.58 to 18.15 for 940 nm, which aligns well with reported clinical ranges.^11^ The relationship between %Mod and systolic pressure is slightly non-linear for both wavelengths [Fig. 11(c)], yet the 660-nm curve shows a mean slope almost twice that for 940 nm. This agrees with prior findings that %Mod tends to be greater for the wavelength where blood absorption is higher.^57^^,^^66^ Overall, these results indicate that our approach has the potential to generate realistic PPG waveforms over a clinically relevant range of %Mod values when used with a finger pulse oximeter sensor.

**Fig. 12:**
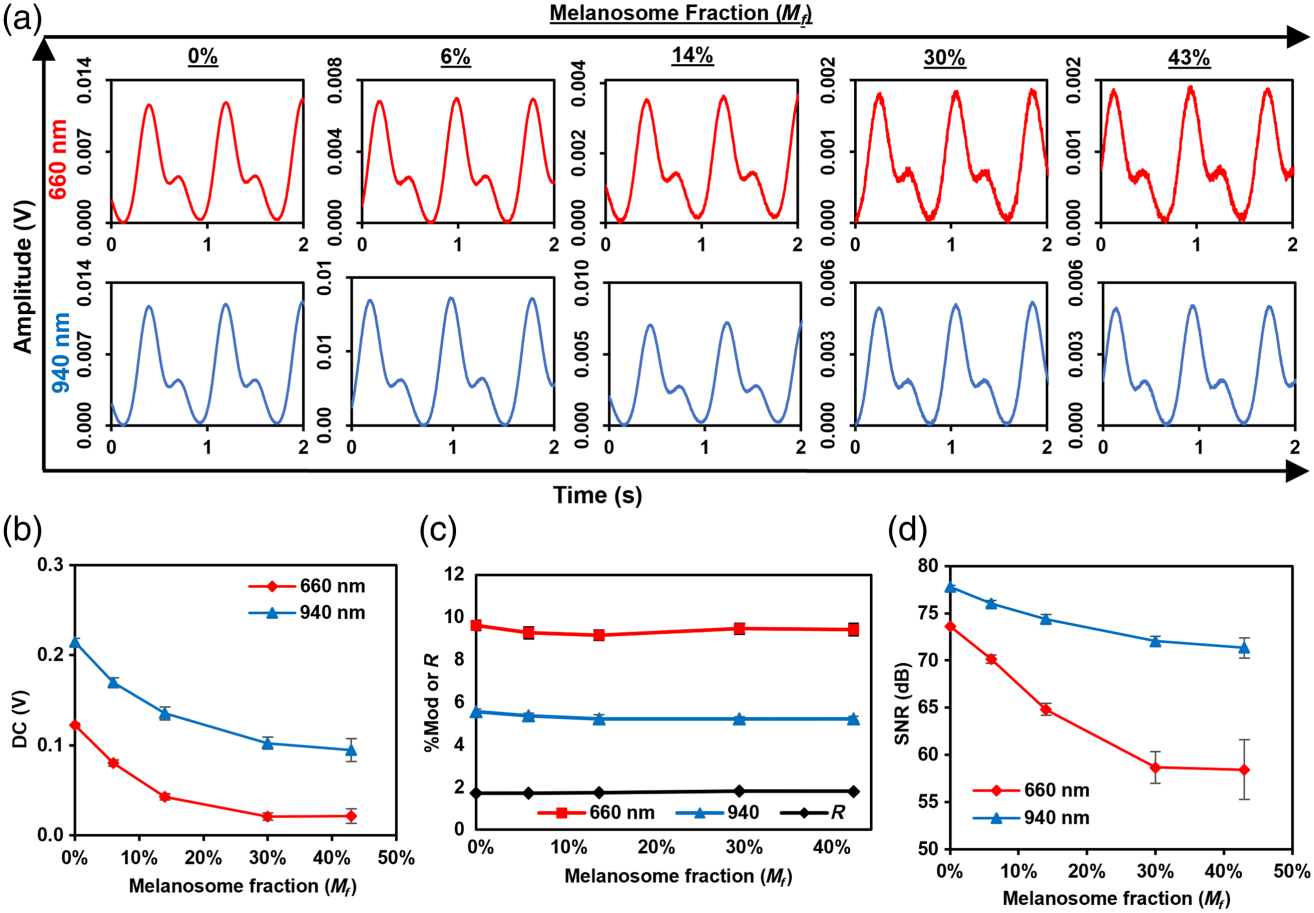
Effect of simulated melanosome volume fraction ($M_{f}$) in a single phantom layer applied to the finger phantom. (a) PPG signal generated at 660 and 940 nm versus $M_{f}$. (b) Average DC component values in finger phantom versus $M_{f}$ at 660 and 940 nm. (c) %Mod or R versus $M_{f}$ at 660 and 940 nm. (d) SNR of PPG signal generated at 660 and 940 nm versus $M_{f}$.

A key application of our flow phantom is to study the effect of skin pigmentation. As a preliminary step toward this goal, we tested the phantom with epidermis-simulating phantom layers of $M_{f}=0\%$, 6%, 14%, 30%, and 43%. The layers were placed on top of the phantom adjacent to the pulse oximeter source. PPG waveforms at 660 and 940 nm for 120/80 mmHg [Fig. 12(a)] show increased high-frequency noise relative to the pulsatile amplitude at the two highest $M_{f}$ levels for 660 nm, which is likely due to a stronger overall reduction of signal [Fig. 12(b)]. The DC signal level decreased with $M_{f}$, as expected, with the decay at 660 nm being much stronger than that for 940 nm [Fig. 12(b)], due to spectral variations in $\mu_{a}$ and $\mu_{s}^{\prime }$. Changes in %Mod as a function of $M_{f}$ were also found, with a decrease of 6.3 % at 660 nm and 7.7% at 940 nm [Fig. 12(c)]. PPG waveform signal-to-noise ratio (SNR) decreased monotonically with $M_{f}$ by 15.2 dB for 660 nm and 6.5 dB for 940 nm, with weaker changes at high $M_{f}$ values [Fig. 12(d)].

To validate the functionality of the finger phantom setup, two clinical devices (Nonin and Masimo) were used to measure the SPO_2_ levels of India ink, approximating blood at ${\mathrm{SaO}}_{2}=70\%$.^57^ The clinical devices displayed ${\mathrm{SpO}}_{2}$ values in the range of 62% to 66% for $M_{f}=0\%$ (Fig. S7 in the Supplementary Material), which aligns well with the expected value. As $M_{f}$ was varied, the ${\mathrm{SpO}}_{2}$ level for the Nonin device remained at a consistent level in the 65.7 to 66 range. The Masimo device exhibited a gradual decrease in ${\mathrm{SpO}}_{2}$, from 63.0% to 60.0%. Neither device showed the small positive bias in ${\mathrm{SpO}}_{2}$ that has been shown in clinical studies.

## 4 Discussion

Fabrication of tissue-mimicking phantoms for pulse oximetry testing requires consideration of a wide variety of factors. A biologically relevant finger phantom should possess realistic mechanical and optical properties to be able to interrogate anatomical and physiological factors that may impact pulse oximeter performance.^29^^,^^42^ Biorelevant mechanical properties will help to ensure that PPG waveforms generated with the phantom material are also realistic. We have also attempted to achieve a degree of realism in finger architecture with fluidic channels. However, an optimal phantom design should account for both the desire for realism and the need for fabrication approaches that are sufficiently simple and reproducible, thus enabling uniformity in testing. The resulting phantoms will provide unique tools for improving a basic understanding of the light-tissue interaction phenomena underlying pulse oximetry and the factors that impact device performance, including skin pigmentation, signal quality, and system design.

Previous studies used glass, plastic, and silicone-based materials to create finger phantoms which had limited ability to mimic tissue elastic modulus and hardness. We have explored silicone-based materials that can be tuned to match the mechanical properties of soft biological tissues. Low PDMS curing agent concentrations provided desirable elastic modulus values, yet increased surface adhesion caused difficulty in handling PDMS samples. This problem was not seen for Ecoflex samples, particularly formulations Ecoflex Mix 20 to 30 and 00 to 30, which best matched the desired material properties.^42^ We observed that Ecoflex possesses lower elastic modulus values, ranging from 65 to 186 kPa, which is similar to that of the subcutaneous tissue and epidermis.^43^ It is also worth noting that PDMS sample modulus values ranging from 682 to 2734 kPa are similar to finger artery and coronary artery values.^67^^,^^68^ The higher modulus range of 682 to 6059 kPa may be suitable to mimic coronary arteries, for example, as healthy and atherosclerotic arteries range from 1550 to 4530 kPa.^68^ The tunability of mechanical properties indicates the strong potential of these materials as physical phantoms for PPG-based devices.

Phantom compliance was also analyzed to ensure that biorelevant changes in blood volume can be achieved to enable the modulation of light absorption necessary to form PPG signals. As internal channel pressure was increased from 0 to 180 mmHg, channel diameters increased by up to 184.5%, thus enabling tests that mimic a range of vessel types, from radial and brachial arteries which expand ∼1.8%,^69^ to finger arteries which expand up to ∼85%.^70^ In a prior study, radial expansion in PDMS was found to be linear with pressure;^29^ however, our results indicated that Ecoflex 00 to 20 and Ecoflex Mix 20 to 30 showed exponential radial expansion with pressure. Although it is unclear the extent to which our results can be extrapolated to other vessel sizes, similar relationships should hold for the 0.7-mm channels used in compliance testing and the 1-mm-diameter channels of the finger phantom. The issue of vessel compliance is tied to vessel geometry considerations (e.g., the need to replicate microvasculature) and physiological factors such as age that correlate with vascular hardening. We will address these issues and the difficulties of incorporating these considerations into phantoms in future studies.

In addition, we have obtained data indicating that the optical and mechanical properties and compliance of the Ecoflex materials remain stable for approximately a year [Figs. 6(c) and 6(d) and Figs. S8(a) and S8(b) in the Supplementary Material]. Further assessments of TMM stability are ongoing and will be published in the future as results become available. Testing with a research pulse oximeter demonstrated the ability to generate biological PPG waveforms with a wide range of %Mod. Simulated systolic and diastolic pressures were tuned to vary %Mod across the clinically relevant range, down to levels that have been shown to cause errors in subjects with high pigmentation (%Mod ≤1).^11^ This capability will be critical for assessing the combined effect of pigmentation and signal quality documented by Gudelunas et al.^11^ In the future, we intend to further explore the issue of compliance, how it changes with vessel diameter, and the degree to which variations in compliance are necessary for generating clinically relevant results.

Although the primary purpose of this work was to advance a phantom-based test method, our measurements on pulse oximeters also provided some potentially relevant insights into skin pigmentation effects.^31^ As expected, increasing $M_{f}$ led to substantial reductions in the magnitude of detected PPG signals. Although only minor degradation in the noise levels of these waveforms was apparent in Fig. 12(a), the quantitative impact on SNR was significant [Fig. 12(d)]. Spectral dependence of signal quality degradation—due to differential absorption by melanin—was also apparent, with a 15-dB decrease in SNR at 660 nm but only a 5-dB decrease at 940 nm. Perhaps more significantly to ${\mathrm{SpO}}_{2}$ measurement errors, our results identified a small but consistent decrease in %Mod with $M_{f}$ [Fig. 12(c)]. This indicates that normalization of the “AC” signal to the “DC” component does not completely mitigate the effect of skin pigmentation. However, when the ratio of ratios was calculated, variations with $M_{f}$ appeared to resolve [Fig. 12(c)]. It is worth noting that variations in both %Mod and R with $M_{f}$ have been documented in modeling work by our group^12^ and others.^3^^,^^8^ Experimental validation of a correlation between R and $M_{f}$ would provide critical evidence that a pulse oximeter’s calibration curve and thus ${\mathrm{SpO}}_{2}$ errors are pigmentation-dependent. In the future, we will implement phantoms that incorporate blood-filled channels to further explore evidence for this correlation. Meanwhile, during the validation of the basic functionality of finger phantom, clinical pulse oximeters never showed a positive bias in ${\mathrm{SpO}}_{2}$ compared with other clinical studies. The reason for this discrepancy is not clear but may be due to the use of a non-blood fluid and/or the relatively high %Mod ranging from 4.1 to 4.9 at which testing was performed. Nevertheless, these preliminary results demonstrate the viability of our phantom approach, as it can be read by clinical devices, and ${\mathrm{SpO}}_{2}$ outputs are in an expected range and some variation with $M_{f}$ was detected. Future parametric studies with a final blood-filled phantom approach promise greater elucidation of pulse oximeter performance and its variation with pigmentation.

In addition to developing pulse oximeter phantoms, secondary contributions of this work include a demonstration of approaches that researchers can use to facilitate phantom optimization. For example, our data showed that durometry offers a simpler alternative to tensile testing for silicone TMM selection. We also implemented a novel approach to TMM compliance measurement using OCT imaging, which can perform non-invasive measurements in turbid phantoms, unlike previously described methods.^29^ In the future, it may also be possible to implement OCT for dynamic measurements during channel pulsation. Finally, our use of a fingerclip sensor similar to clinical devices to perform this type of testing appears novel. As clinical devices typically do not provide quantitative PPG waveform data, and none provides this data for the red channel—which is most sensitive to melanin content—we believe such a research instrument will be very useful for elucidating pulse oximeter performance.

## 5 Conclusion

To overcome the limitations of prior pulse oximeter bench test methods, we have developed an approach based on novel silicone tissue-mimicking finger phantoms as part of a pulsatile fluid system. This system provides a balance between birelevance and simplicity to facilitate broad implementation. We identified an optimal TMM formulation with biorelevant optical and mechanical properties and demonstrated the construction of finger-shaped flow phantoms that allow the generation of PPG waveforms. By adjusting the input pressure waveform, it was possible to generate biorelevant PPG waveforms over a wide range of pulsatile modulation levels, and variations in waveform magnitude were also demonstrated by adding superficial layers simulating pigmented epidermis. Our preliminary investigation with clinical pulse oximeters showed they were able to generate ${\mathrm{SpO}}_{2}$ measurements using this approach, albeit at low ${\mathrm{SpO}}_{2}$ levels given the nigrosin solution, so modulation of saturation levels should be possible in the future using bovine blood, as in our prior cerebral oximetry phantom studies.^31^ Overall, our results indicate that this phantom-based approach has a range of useful features that will enable more realistic testing than previously achievable in bench tests for pulse oximetry.

## Supplementary Material



## Data Availability

Data produced in this study are available upon reasonable request.
