# Supplementary material for: Development and characterization of silicone-based tissue phantoms for pulse oximeter performance testing
Source: J Biomed Opt. 2025 Jan 7;29(Suppl 3):S33314. doi: 10.1117/1.JBO.29.S3.S33314 (PMC11706025; doi:10.1117/1.JBO.29.S3.S33314)
Supplement: Supplementary file 1 [file JBO_029_S33314_SD001.docx]

**Supplemental Material**

**Title: Development and characterization of silicone-based tissue phantoms for pulse oximeter performance testing**

Anant Bhusal,^a,b*^ Masoud Farahmand,^b^ Md Sadique Hasan,^b^ Sandhya Vasudevan,^b^ William C Vogt,^b^ Bryan Ibarra,^b^ Sandy Weininger,^b^ Christopher G. Scully,^b^ X. Frank Zhang,^a^ Yu Chen,^a^ T. Joshua Pfefer,^b^

^a^Department of Biomedical Engineering, University of Massachusetts, Amherst, MA 01003 USA

^b^Center for Devices and Radiological Health, Food and Drug Administration, 10903 New Hampshire Ave., Silver Spring, Maryland 20993, USA

**Table S1.** Hardness of Ecoflex 00-30 cured at room temperature and after heating at 80^o^C for 4 hours.

| **Hardness Scale: Shore OO** | | | | **Average** | **Stdev** |  |
| --- | --- | --- | --- | --- | --- | --- |
|  |  |  |  |  |  |  |
| **Ecoflex 00-30** | **Reading #1** | **Reading #2** | **Reading #3** |  |  |  |
| Before Heat Treating | **31** | **32** | **33** | **32** | **1.00** |  |
| Heat Treating (4 hours at 80^o^C) | **31** | **33** | **33** | **32.3** | **1.15** |  |

**Table S2.** List of materials used to create epidermis-simulating phantom.

| **Materials** |  | **Company** |
| --- | --- | --- |
| Nigrosin (water soluble) | CAS Number: 8005-03-6 | Sigma-Aldrich, St. Louis, MO |
| Titanium (IV) oxide, anatase | CAS Number: 1317-70-0 | Sigma-Aldrich, St. Louis, MO |
| Sylgard 184 Silicone Elastomer |  | Dow Corning, Midland, MI |
| Nail Polish Remover | Beauty 360, Original | CVS, Woonsocket, RI |

**Table S3.** List of materials used for pulsatile fluid testing of pulse oximeter phantoms.

| **Item** | **Model** | **Manufacturer Information** |
| --- | --- | --- |
| Polyvinyl Chloride Tubing | ND-100-65, Tygon® | Saint-Gobain Corporation, Courbevoie, France |
| Pressure Pulse Generator | PPG-601A | Flometrics Inc., Carlsbad, CA |
| Pulse Oximeter | AFE4490SPO2EVM | Texas Instruments, Inc., Dallas, TX |
| Pressure Recording Unit | Millar PCU-2000 | Millar, Houston, TX |
| Pressure Catheters | MPR-500 Mikro-tip pressure catheter | Millar, Houston, TX |
| National Instruments Data Acquisition Interface | cDAQ-9174 | National Instruments Corp., Austin, TX |
| Voltage Output Module | NI-9263 | National Instruments Corp., Austin, TX |


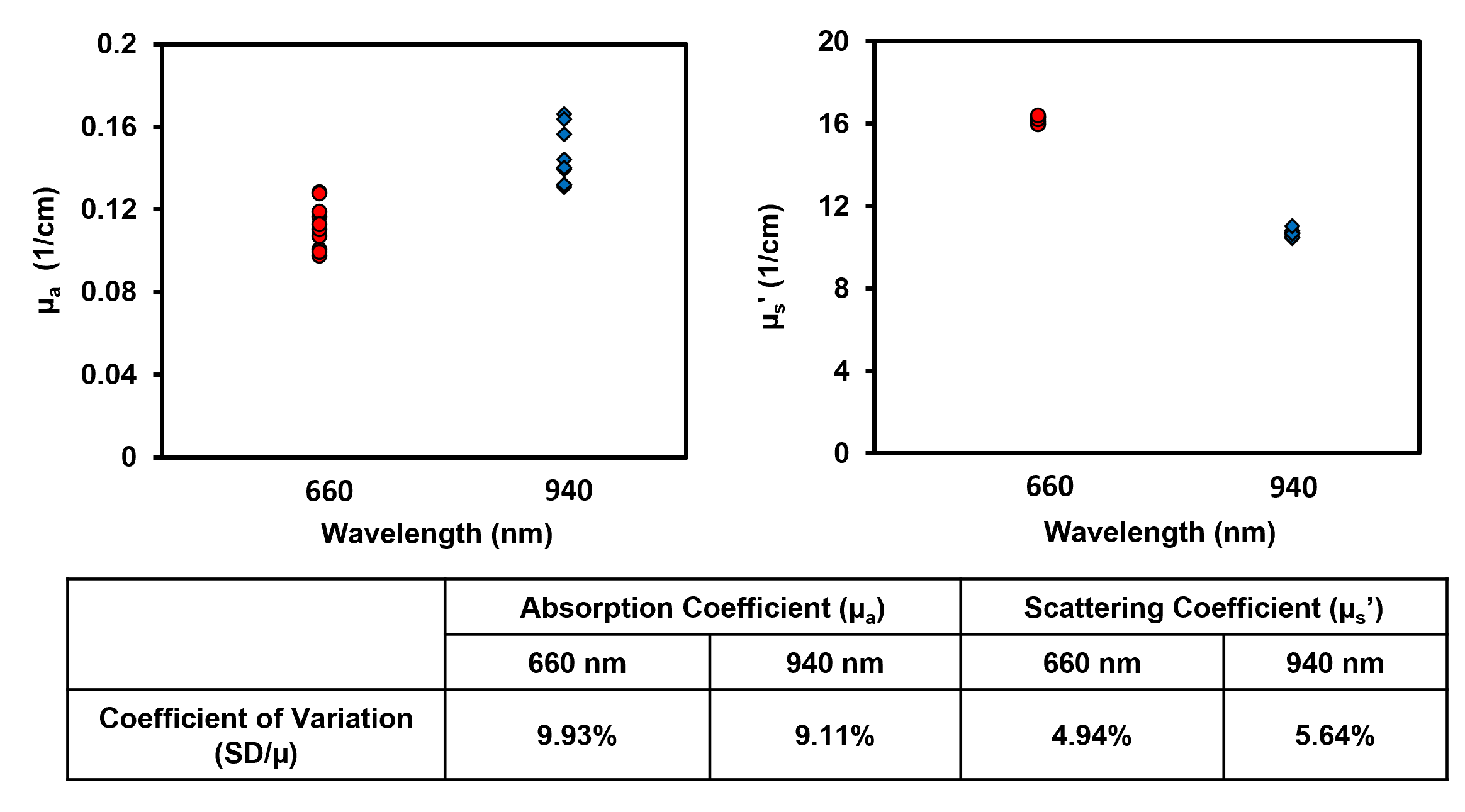


**Figure S1.** Measurement for accuracy of the IAD system using Ecoflex 00-30 with 1.25 mg/g TiO_2_ samples: (a) Absorption Coefficient (µ_a_) at 660nm and 940 nm, and (b) Reduced Scattering Coefficient – (µ_s_’) at 660nm and 940nm.


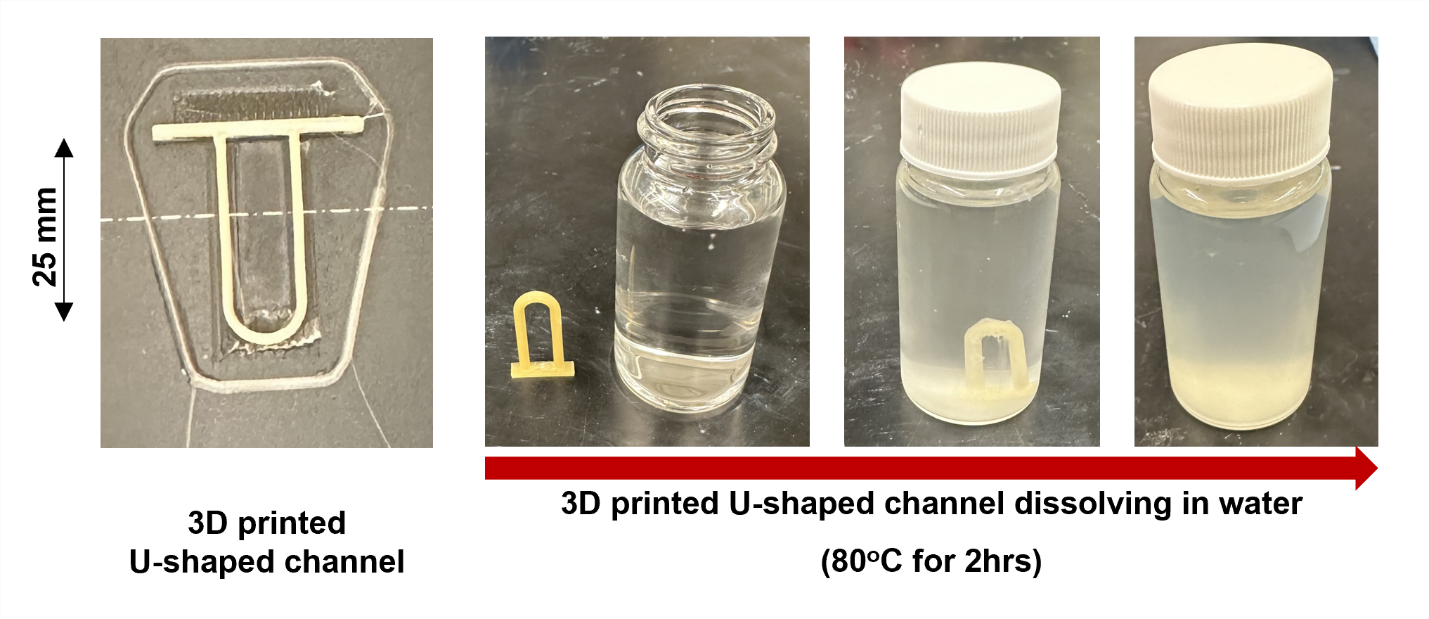


**Figure S2**. PrimaSelect PVA+ 3D printing material solubility test.


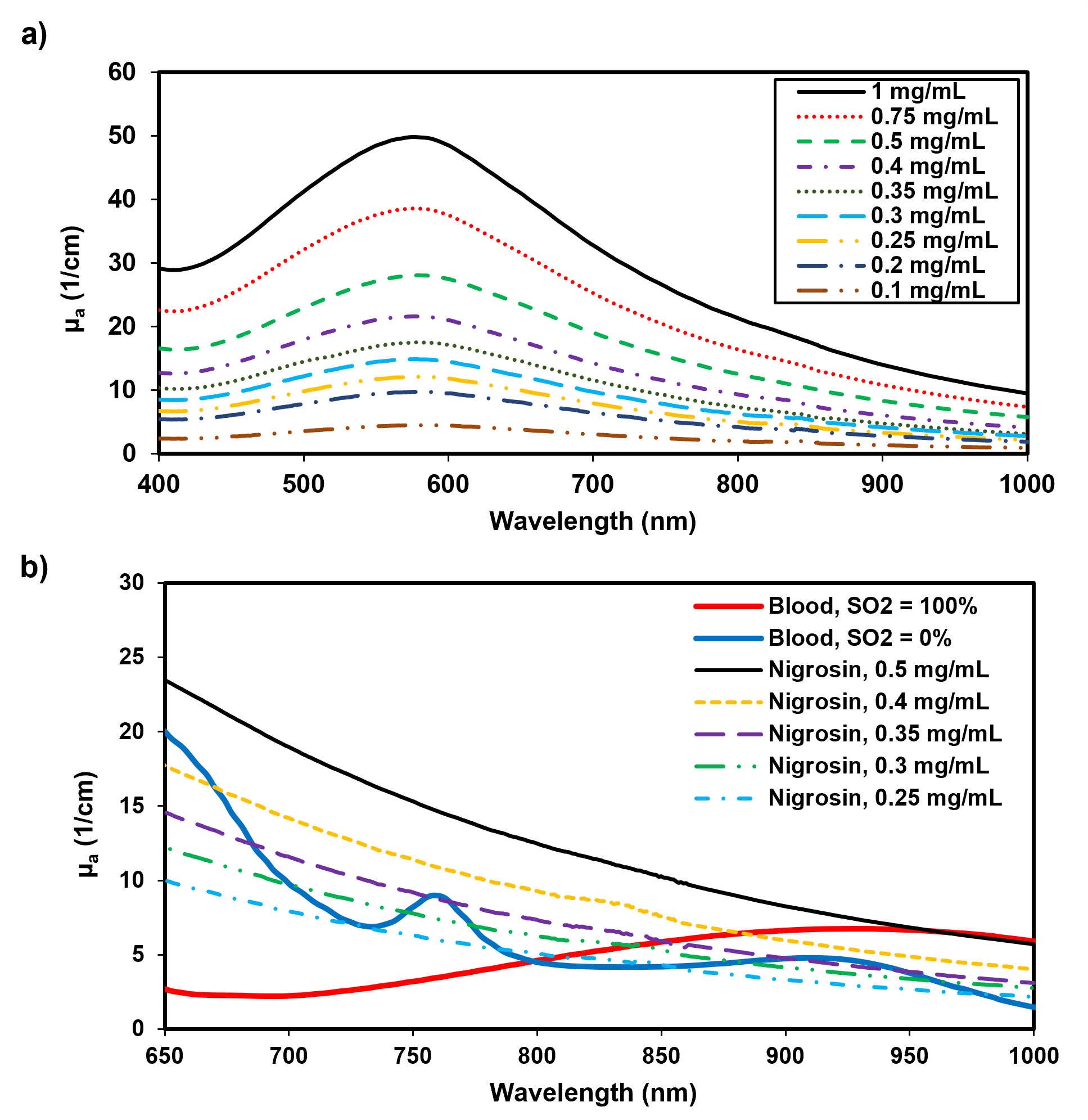


**Figure S3**. (a) Absorption coefficient spectra of water-soluble nigrosin in DI water at different concentration, and (b) Absorption coefficient spectra of water-soluble nigrosin in DI water vs. reported data for blood [51].


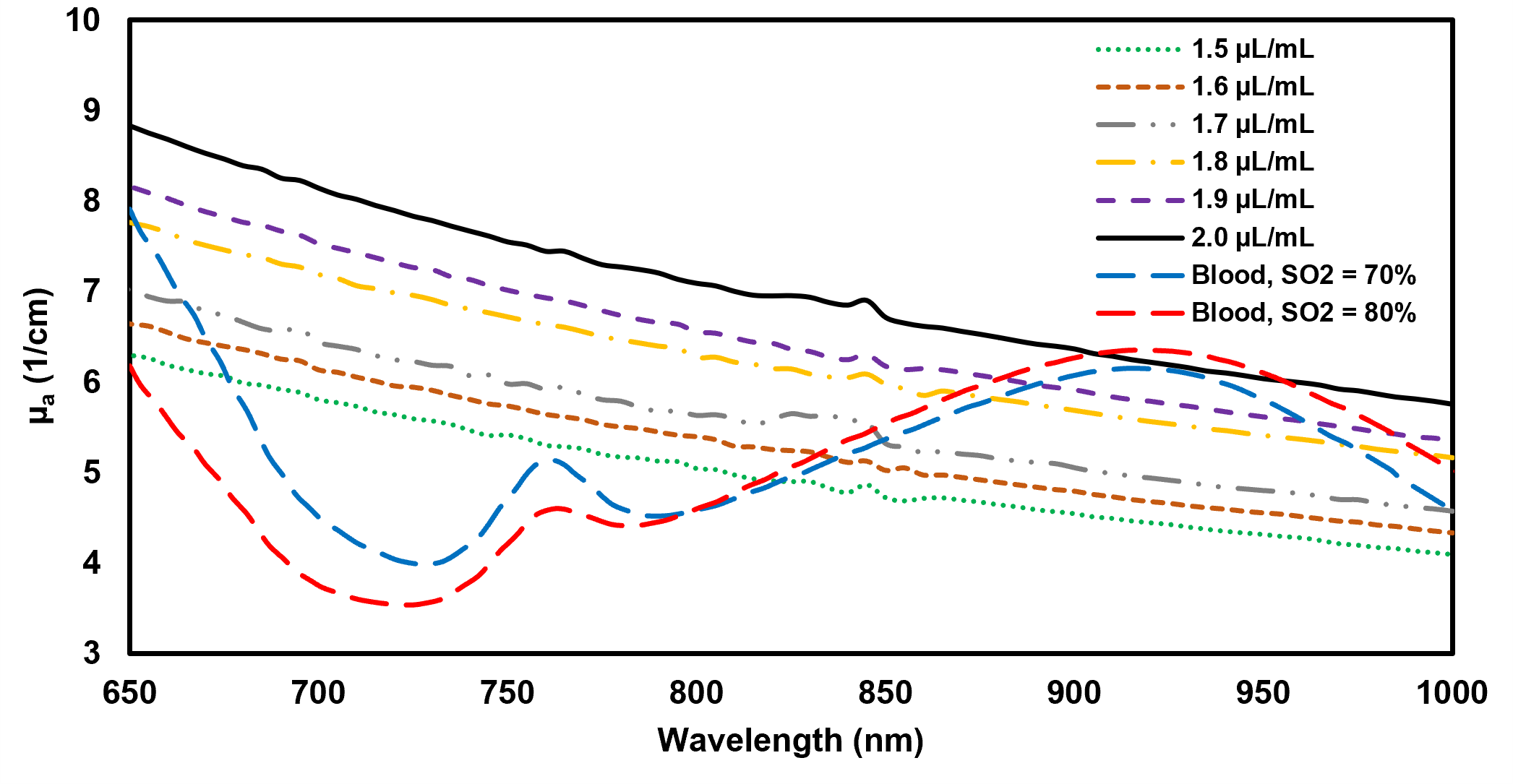


**Figure S4.** Comparison absorption coefficient of India Ink in DI water at different concentration vs. reported data for blood [51].


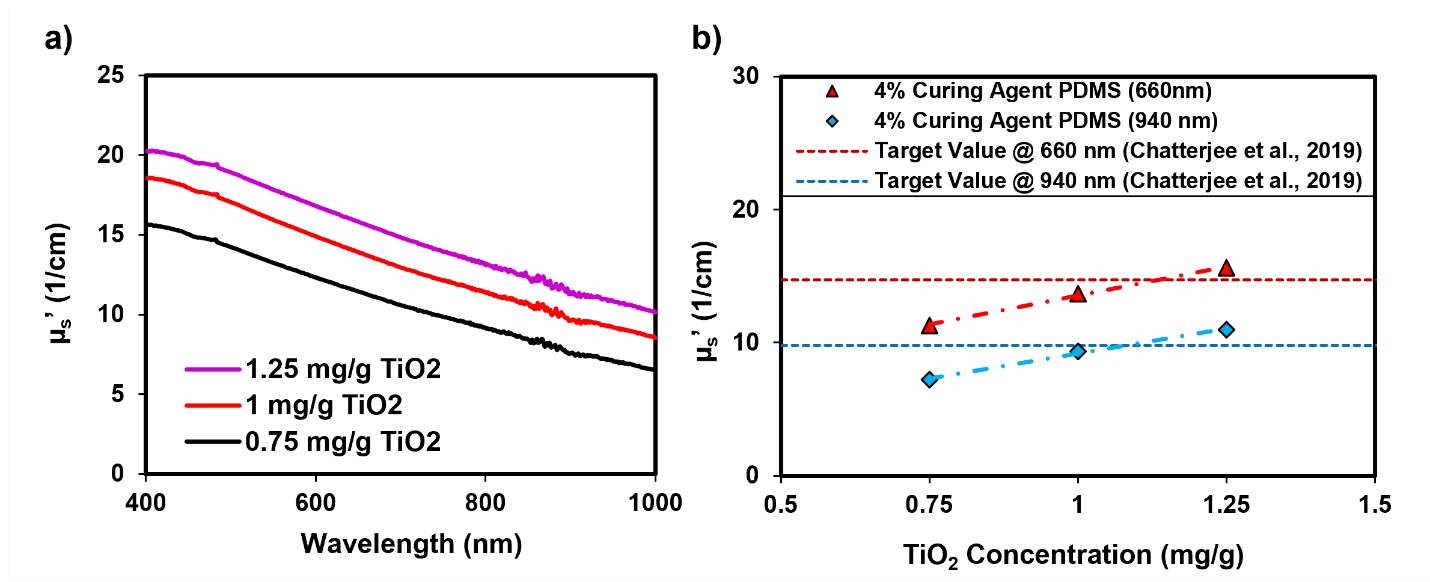


**Figure S5**. (a) Reduced scattering coefficient spectra from 400 nm to 1000 nm vs. TiO_­2_ concentration in PDMS (4% curing agent), and (b) reduced scattering coefficient of TiO_2_ mixed with PDMS (4% curing agent) mixed at 660 nm, and 940 nm.


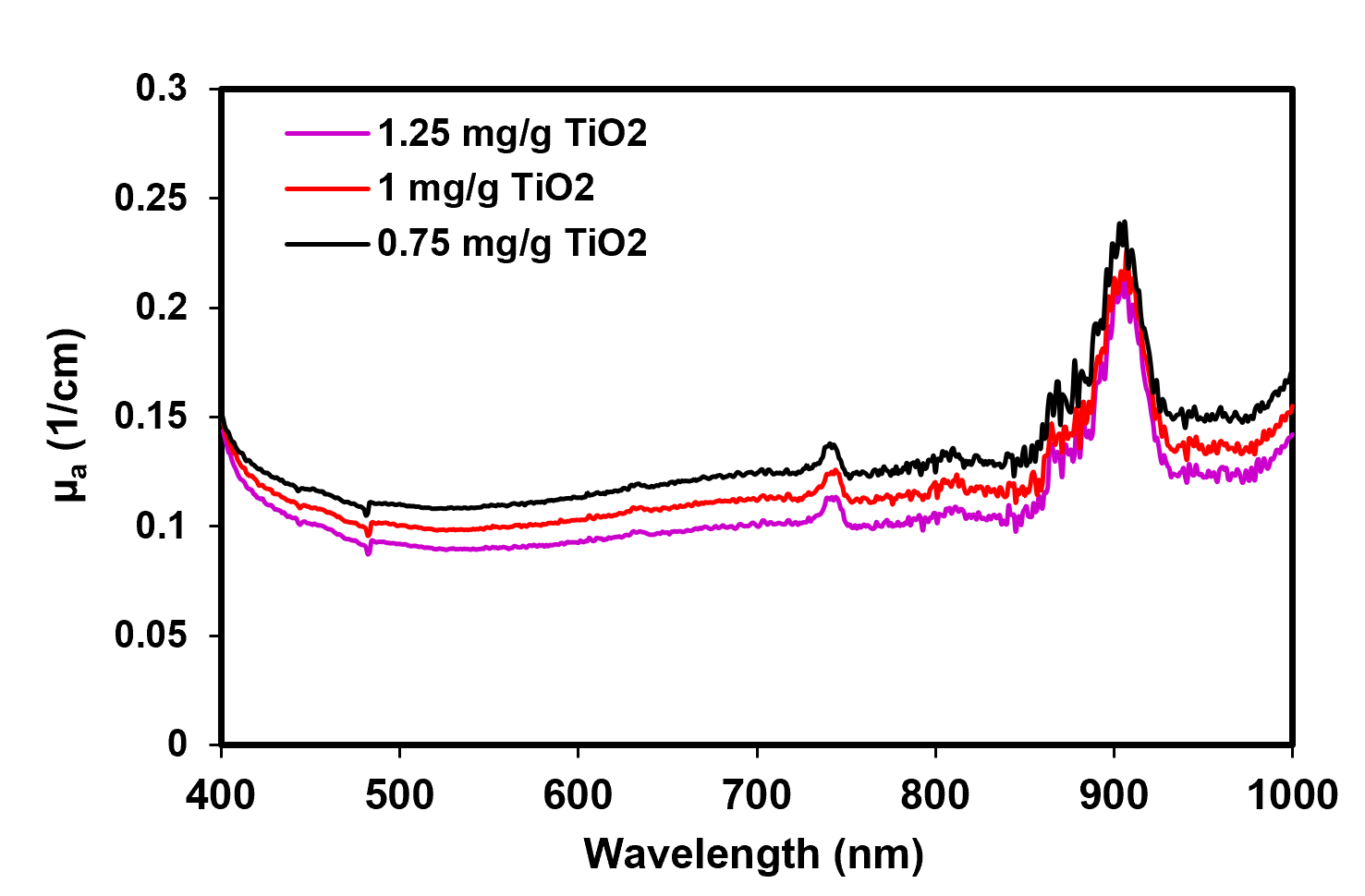


**Figure S6**. Absorption Coefficient of Ecoflex 00-30 mixed with TiO2 at different concentration (0.75, 1, and 1.25 mg/gm) at wavelength ranging from 400 nm to 1000 nm.


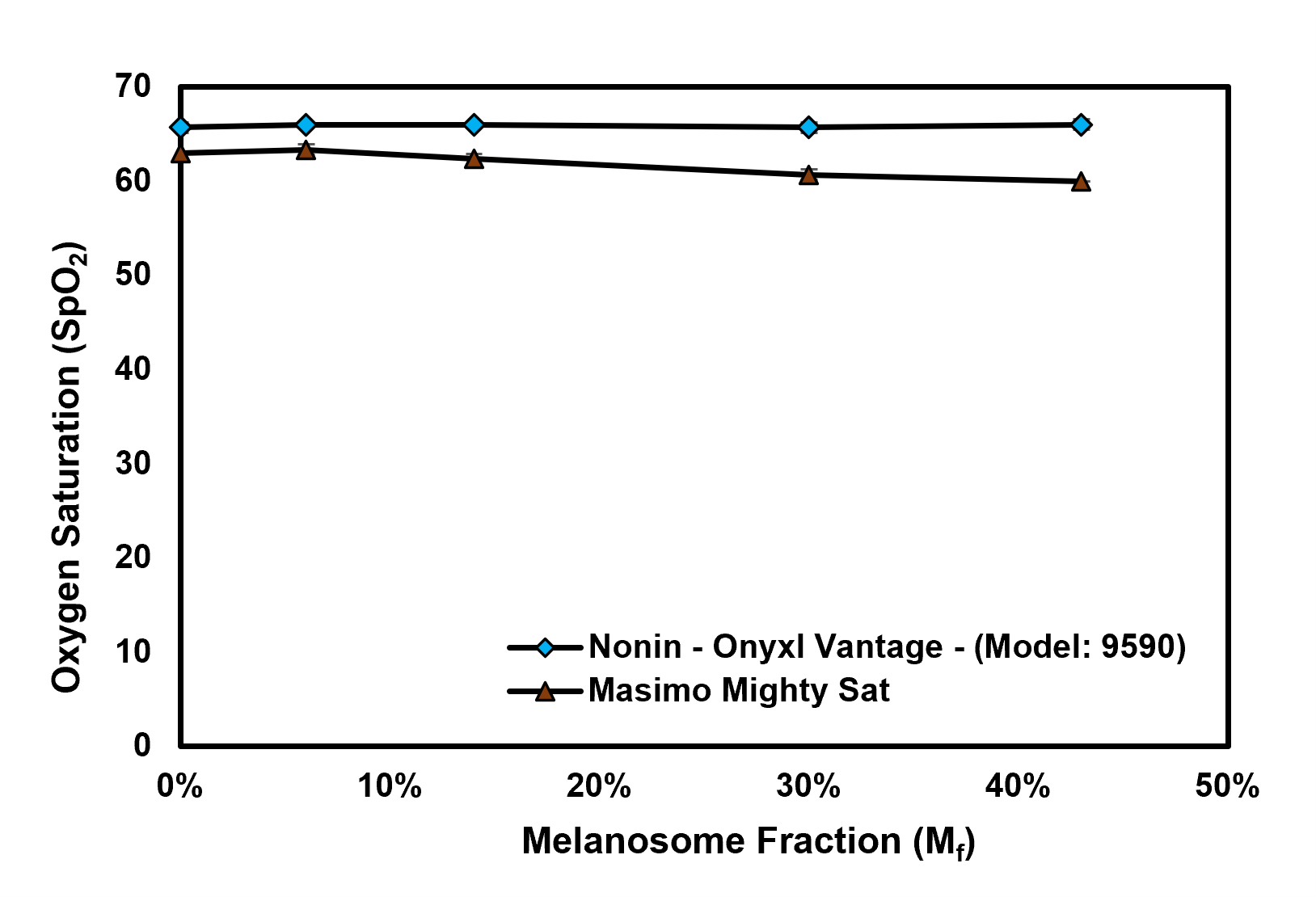


**Figure S7.** Measurement of SpO2 using two different pulse oximeters (Nonin and Masimo) with different melanosome fraction (M_f_).


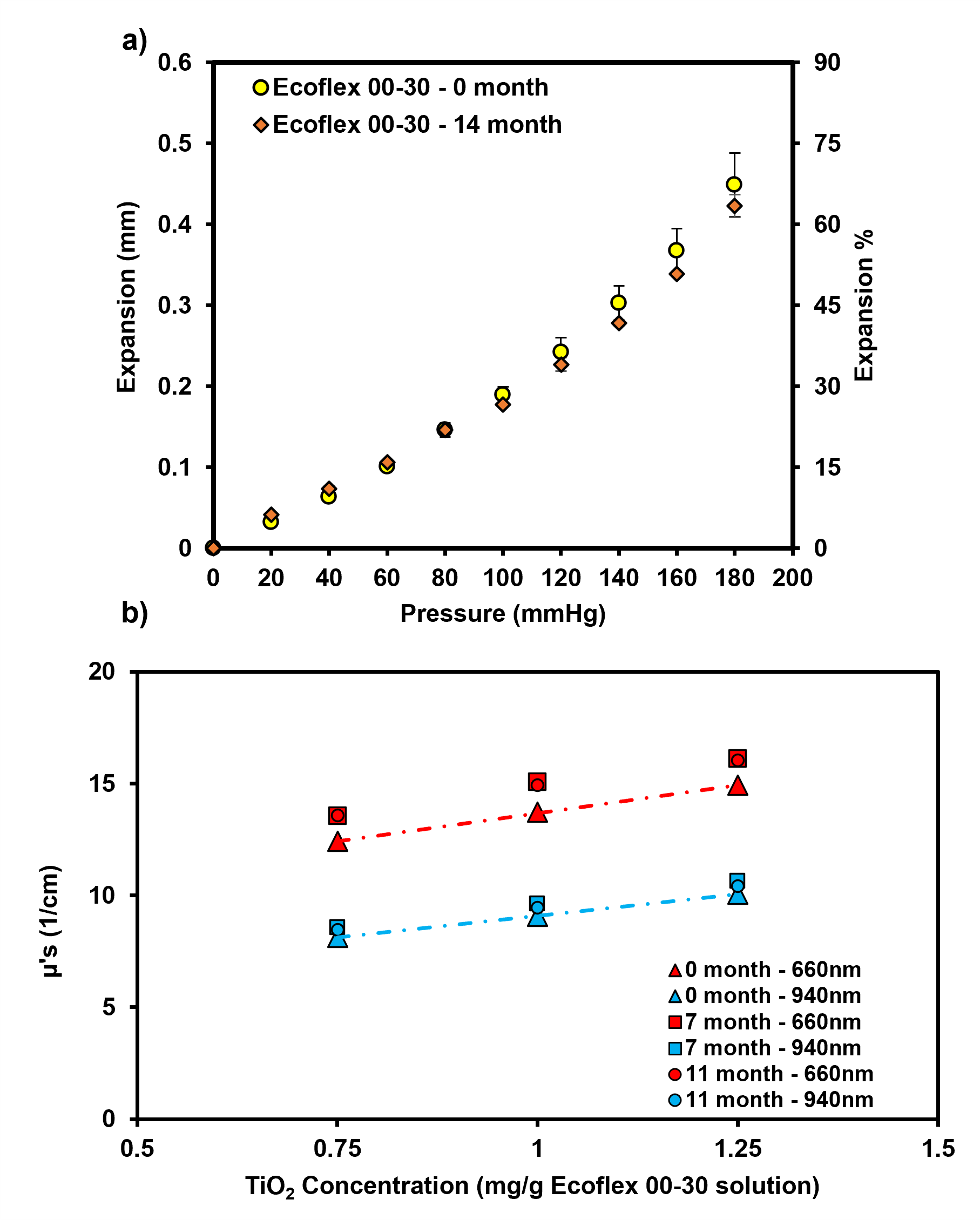


**Figure S8.** (a) Expansion of straight channels (diameter: 0.71 mm) in Ecoflex 00-30 as a function of pressure immediately after fabrication and after 14 months, and (b) Reduced Scattering Coefficient of Ecoflex 00-30 with TiO2 at different concentration (0.75, 1, and 1.25 mg/g) at wavelength ranging from 400 nm to 1000 nm till 11 months after fabrication.
